# Focal adhesion kinase plays a dual role in TRAIL resistance and metastatic outgrowth of malignant melanoma

**DOI:** 10.1038/s41419-022-04502-8

**Published:** 2022-01-12

**Authors:** Greta Del Mistro, Shamala Riemann, Sebastian Schindler, Stefan Beissert, Roland E. Kontermann, Aurelien Ginolhac, Rashi Halder, Luana Presta, Lasse Sinkkonen, Thomas Sauter, Dagmar Kulms

**Affiliations:** 1 Experimental Dermatology, Department of Dermatology, TU-Dresden, 01307 Dresden, Germany; 2 National Center for Tumor Diseases Dresden, TU-Dresden, 01307 Dresden, Germany; 3 Institute of Cell Biology and Immunology and Stuttgart Research Centre Systems Biology, University of Stuttgart, 70569 Stuttgart, Germany; 4 Department of Life Sciences and Medicine, University of Luxembourg, Belvaux, 4367 Luxembourg; 5 Luxembourg Centre for Systems Biomedicine, University of Luxembourg, Belvaux, 4367 Luxembourg

**Keywords:** Metastasis, Target identification

## Abstract

Despite remarkable advances in therapeutic interventions, malignant melanoma (MM) remains a life-threating disease. Following high initial response rates to targeted kinase-inhibition metastases quickly acquire resistance and present with enhanced tumor progression and invasion, demanding alternative treatment options. We show 2^nd^ generation hexameric TRAIL-receptor-agonist IZI1551 (IZI) to effectively induce apoptosis in MM cells irrespective of the intrinsic BRAF/NRAS mutation status. Conditioning to the EC_50_ dose of IZI converted the phenotype of IZI-sensitive parental MM cells into a fast proliferating and invasive, IZI-resistant metastasis. Mechanistically, we identified focal adhesion kinase (FAK) to play a dual role in phenotype-switching. In the cytosol, activated FAK triggers survival pathways in a PI3K- and MAPK-dependent manner. In the nucleus, the FERM domain of FAK prevents activation of *wt*p53, as being expressed in the majority of MM, and consequently intrinsic apoptosis. Caspase-8-mediated cleavage of FAK as well as FAK knockdown, and pharmacological inhibition, respectively, reverted the metastatic phenotype-switch and restored IZI responsiveness. FAK inhibition also re-sensitized MM cells isolated from patient metastasis that had relapsed from targeted kinase inhibition to cell death, irrespective of the intrinsic BRAF/NRAS mutation status. Hence, FAK-inhibition alone or in combination with 2nd generation TRAIL-receptor agonists may be recommended for treatment of initially resistant and relapsed MM, respectively.

## Introduction

Targeted therapies as well as immune checkpoint inhibition currently offer a long-term survival to ~30% of patients with metastatic melanoma (MM). Activating mutations of the serine-threonine kinases NRAS (*mut*NRAS) or BRAF (*mut*BRAF) are key drivers of tumor growth through constitutive activation of Mitogen-Activated Protein Kinase (MAPK) pathways involving RAF-MEK-ERK and PI3K-AKT-mTOR, respectively [1–3]. Combination of mutation-specific BRAF inhibitors, and MEK inhibitors show high response rates on *mut*BRAF MM [4], however, most patients, acquire resistance resulting in tumor relapse [5, 6]. No targeted therapeutics exist to date to effectively treat *mut*NRAS MM [7], and response rates to immune checkpoint inhibition remain rather low [8]. Hence, ~70% of patients still succumb to the disease due to enhanced regrowth of treatment-resistant metastases.

Metastatic outgrowth involves loss of cellular adhesion, and invasive growth through the extracellular matrix (ECM) [9]. Integrins, a family of cell-surface adhesion receptors are composed of α- and β-subunits that mediate adhesion to the ECM [10, 11]. Upon activation, integrins convey intracellular processes through activation of focal adhesion kinase (FAK/PTK2) and SRC kinase family members to trigger anchorage-independent cell survival, proliferation, and migration [12–14]. Activation of FAK requires a conformational change to uncover its kinase domain. Displacement of the N-terminal FERM domain is facilitated upon interaction with the cytosolic domains of β-integrins, allowing activation of its kinase function through autocatalytic phosphorylation of Tyr397 [15]. In the cytosol, activated FAK interacts with a variety of cytoskeletal proteins and upstream kinases including SRC, PI3K, and PDK1 to mediate downstream activation of RAF-MEK-ERK and PI3K-AKT-(mTOR) signaling pathways [16, 17]. A fraction of FAK, however, translocates into the nucleus where its FERM domain interacts with the N-terminal domain of wildtype p53 (*wt*p53) and MDM2 to facilitate p53 degradation thereby preventing apoptosis [17–19]. Accordingly, FAK may play an important role in metastatic progression and apoptosis inhibition in *wt*p53-expressing MM.

Aiming to identify alternative treatment options for primary and relapsed MM we recently showed activation of the tumor necrosis factor-related apoptosis-inducing ligand (TRAIL)-receptor with a 2^nd^ generation hexameric TRAIL-receptor agonist (IZI1551 [20, 21]), to be superior in subjecting *mut*BRAF MM cells to cell death compared to combined *mut*BRAF/MEK inhibition [22]. In the present study we discovered that acquired IZI1551-resistance of *mut*BRAF and *mut*NRAS MM cells due to long-term exposure to the EC_50_ dose of IZI1551 coincides with an enhanced metastatic phenotype, and identified FAK to play a dual role in the observed phenotype-switch.

## Results

### Conditioning to IZI5 renders MM cells resistant to TRAIL-receptor-agonist-induced apoptosis and causes expansion of 3D spheroids

**Fig. 1:**
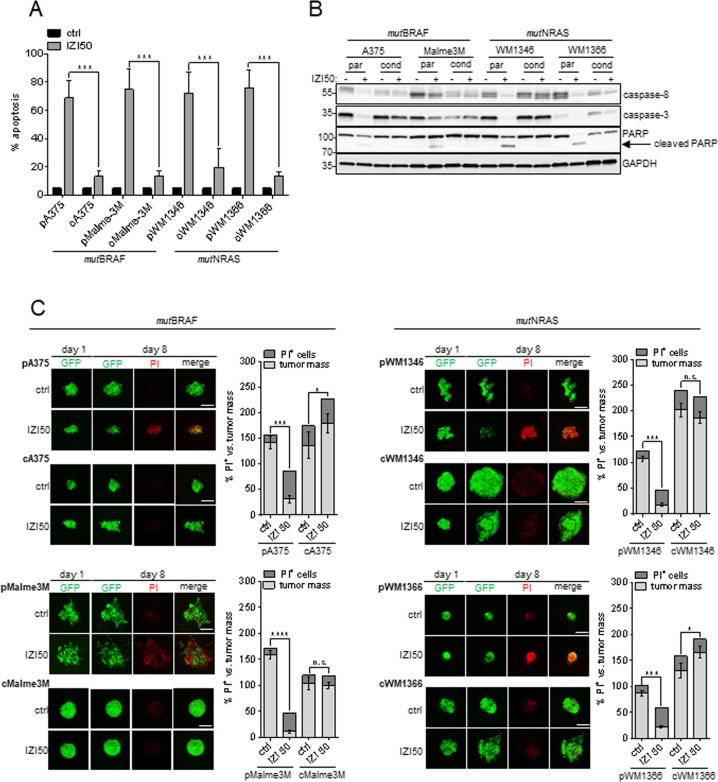
Conditioning to IZI5 renders MM cells resistant to TRAIL-receptor-agonist-induced apoptosis and causes expansion of 3D spheroids. **A** Apoptosis induction was monitored 16 h after stimulation of parental (p) and IZI5-conditioned (c) BRAF-mutated (*mut*BRAF) A375 and Malme3M as well as NRAS-mutated (*mut*NRAS) WM1346 and WM1366 MM cells with IZI50 using a Cell Death Detection ELISA (CDDE) (*n* = 3: ***p ≤ 0.001). **B** Processing of caspase-8, caspase-3, and PARP was assessed by Western-blot analysis with GAPDH as loading control. One representative out of three independently performed experiments is shown. **C** GFP-expressing green fluorescent spheroids consisting of parental (p) and IZI5-conditioned (c) melanoma cells were individually embedded into 3D dextran-based gel-matrices and stimulated with IZI50 every other day over 8 days (scale bar = 200 µm). At day eight cell death was visualized by addition of 6.7 µg/ml PI. Confocal images of individual spheroids at day one and eight and the quantification of tumor volume (green) versus tumor death (red) at day eight are shown (*n* = 3: *p ≤ 0.05; ***p ≤ 0.001; n.s. = not significant).

Based on our hypothesis that tumor cells that do not receive the full lethal, but a suboptimal drug dose may survive treatment, undergo phenotype-switching and cause tumor relapse, we conditioned two *mut*BRAF (A375, Malme3M) and two *mut*NRAS (WM1346, WM1366) MM cell lines to the TRAIL-receptor-agonist IZI1551 (IZI), by exposing them to the EC_50_ dose of 5 ng/ml (IZI5; [22]) for 6 months. Subsequent, exposure of cells to a lethal IZI dose of 50 ng/ml (IZI50), exclusively induced apoptosis in parental cells, while protecting the IZI5-conditioned counterparts (Fig. 1A). Accordingly, processing of caspase-8, caspase-3, and PARP only occurred in parental but not in IZI5-conditioned cells (Fig. 1B). Expression of caspase-8 was diminished in IZI5-conditioned cells, implying that only those cells survived conditioning that expressed lower caspase-8 level, and may therefore cause attenuation of IZI50-induced apoptosis [22, 23] (Fig. 1B). Lack of apoptosis induction in response to IZI5-conditioning was confirmed in an in vivo-mimicking 3D setting of GFP-expressing MM spheroids [24] embedded into gel-matrices that allow monitoring of the same spheroid over time. Eight days after continuous IZI50 treatment, tumor mass (green) *versus* % PI-positive (PI^+^) dead cells (red) was quantified relative to corresponding untreated controls. As expected, IZI50 treatment of parental spheroids resulted in a significantly decreased tumor volume, and an increased amount of dead cells (Fig. 1C). In contrast, IZI5-conditioned spheroids hardly showed PI positivity while, strikingly, three of them (cA375, cWM1346, cWM1366) presented with significantly increased volumes, and outgrowth of cells from the spheroid core (Fig. 1C), implying that long-term exposure to the EC_50_ dose of IZI1551, confers treatment resistance, and concomitantly drives tumor regrowth and metastatic outgrowth.

### Conditioning to IZI5 enhances proliferation of *mut*BRAF and *mut*NRAS MM cells

**Fig. 2:**
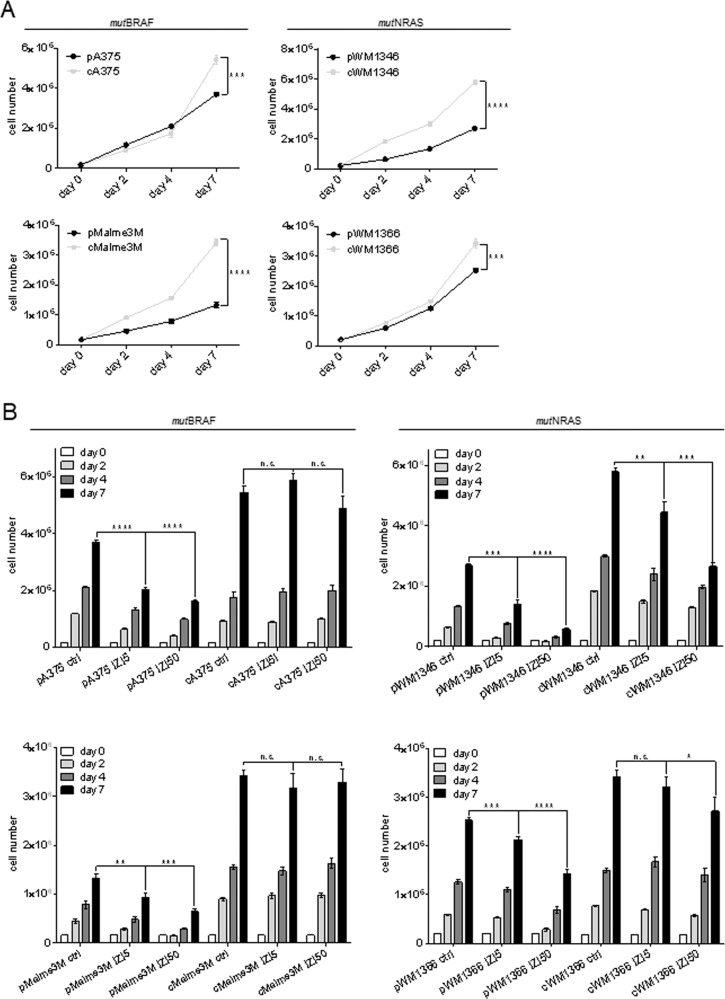
Conditioning to IZI5 enhances proliferation of *mut*BRAF and *mut*NRAS MM cells. **A** Proliferation of unstimulated parental (p) *versus* IZI5-conditioned (c, 5 ng/ml) MM cells was quantified at the indicated time points (***p ≤ 0.001; ****p ≤ 0.0001). **B** Parental (p) and IZI5-conditioned (c) cells were left untreated or stimulated with IZI5 and IZI50, respectively, every other day. Proliferation of cells was quantified at the indicated time points (*n* = 3: *p ≤ 0.05; **p ≤ 0.01; ***p ≤ 0.001; ****p ≤ 0.0001; n.s. = not significant).

Increased spheroid volumes implied that IZI5-conditioned cells proliferated faster. Indeed, untreated IZI5-conditioned cells showed significant higher proliferation compared to the individual parental counterparts (Fig. 2A). Accordingly, treatment with IZI5 significantly decreased proliferation of parental cells, being more pronounced upon IZI50 treatment (Fig. 2B, Fig. S1). In contrast, neither exposure to the EC_50_ nor the lethal IZI dose significantly influenced accelerated proliferation of IZI5-conditioned *mut*BRAF A375 and Malme3M, or *mut*NRAS WM1366 cells. Only proliferation of *mut*NRAS WM1346 cells was diminished, but still as high as of untreated parental cells (Fig. 2B, Fig. S1), indicating that the vital status of IZI5-conditioned cells remains largely unaffected upon exposure to the lethal dose.

### Conditioning to IZI5 enhances clonogenic outgrowth and migration of *mut*BRAF and *mut*NRAS MM cells

**Fig. 3:**
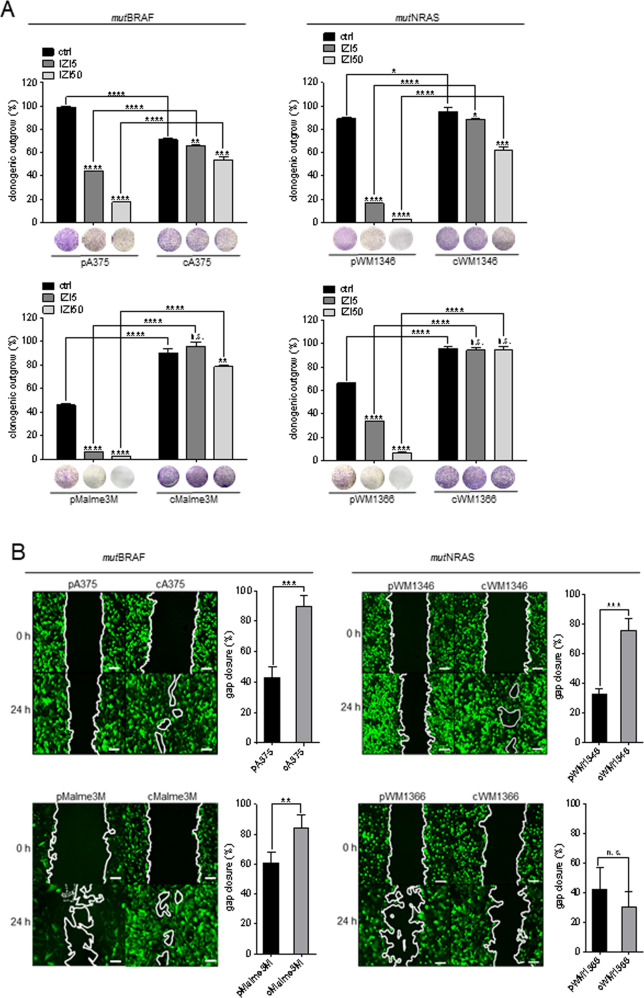
Conditioning to IZI5 enhances clonogenic outgrowth and migration of *mut*BRAF and *mut*NRAS MM cells. **A** Parental (p) and IZI5-conditioned (c) MM cells seeded at 10% confluency were left untreated or stimulated with IZI5 and IZI50, respectively, every other day. After 8 days, colonies were stained with crystal violet, and absorbance of eluted crystal violet at 595 nm was quantified (*n* = 3) (*p ≤ 0.05; **p ≤ 0.01; ***p ≤ 0.001; ****p ≤ 0.0001; n.s. = not significant). **B** Migration of untreated GFP-expressing parental (p) and IZI5-conditioned (c) cells was monitored by scratch assay (scale bar = 100 µm) and gap closure quantified after 24 h (*n* = 3: **p ≤ 0.01; ***p ≤ 0.001; n.s. = not significant).

Clonogenic outgrowth was enhanced in three (Malme3M, WM1346, WM1366) out of four untreated IZI5-conditioned compared to parental cells, and remained significantly less affected upon treatment with IZI5 or IZI50, irrespective of the intrinsic mutation status (Fig. 3A).

In order to monitor the effect of IZI5-conditioning on tumor cell migration we performed scratch assay. After 24 h gap closure was almost complete for GFP-expressing IZI5-conditioned A375, Malme3M and WM1346 cells, whereas only 40–60% of the gap had been invaded by parental cells (Fig. 3B). Surprisingly, IZI5-conditioned WM1366 cells presented with slightly attenuated gap closure compared to parental cells (Fig. 3B).

To this end, we demonstrate that IZI5-conditioned *mut*BRAF and *mut*NRAS MM cells (i) remained resistant against the lethal IZI50 dose, (ii) presented with tumor outgrowth in response to IZI50, and iii) showed enhanced proliferation, clonogenic outgrowth, and migration. Notably, not every MM cell pair showed each of the above-mentioned phenotypes, reflecting the heterogeneity of individual tumor specimens that should be considered during the identification of common druggable targets.

### Transcriptomic analysis identifies FAK as key regulator of the metastatic phenotype of IZI5-conditioned MM cells

**Fig. 4:**
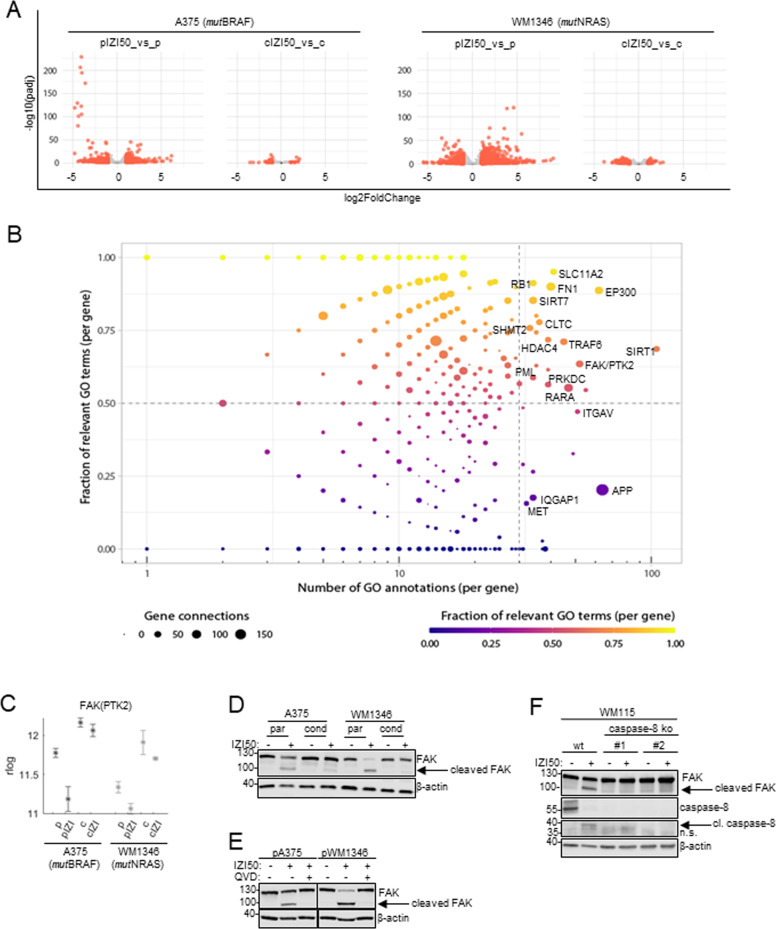
Transcriptomic analysis identifies FAK as key regulator of the metastatic phenotype of IZI5-conditioned MM cells. **A** Parental (p) and conditioned (c) A375 and WM1346 cells remained untreated or where stimulated with IZI50 for six hrs. Subsequently, RNAseq analysis of three biological replicates was performed and gene expression changes presented by vulcano plots. Genes are represented as dots in −log10 (adjusted p-value) versus log2 (fold-change). Differentially expressed genes with adjusted *p*-value ≤ 0.1 and abs (fold-change) ≥ 1 are marked in red. **B** Differentially expressed genes with consistent directionality of change in the parental contexts of both cell lines, but not being differentially expressed in the conditioned contexts (1248 genes), were fed into GeneWalk with default settings. Regulators are represented as dots in ‘Fraction of Relevant GO Terms per Gene’ *versus* ‘Number of GO Annotations per Gene’. **C** Mean expression (rlog counts) of the FAK(PKT2) gene in parental (p) and IZI5-conditioned (c) A375 and WM1346 cells in response to IZI50 treatment for 6 h. **D** Parental (p) and IZI5-conditioned (c) A375 and WM1346 cells were stimulated with IZI50. After 24 h processing of FAK was determined by Western-blot analysis. One representative out of three independently performed experiments is shown. **E** Parental (p) A375 and WM1346 cells were pretreated or not with 5 µM QVD for 1 h. Subsequently cells were stimulated with IZI50 and cleavage of FAK monitored 24 h later by Western-blot analysis. One representative out of three independently performed experiments is shown. **F** Wildtype (wt) and caspase-8 knock out (clones #1 and #2) WM115 melanoma cells were stimulated with IZI50 and the protein/cleavage status of FAK and caspase-8 assessed after 24 h by Western-blot analysis. β-actin served as loading controls. One representative out of three independently performed experiments is shown.

Aiming to identify common molecular changes in *mut*BRAF and *mut*NRAS MM cells that may confer phenotype-switching, we performed RNAseq analysis of parental and IZI5-conditioned IZI50-stimulated cells, and used rlog regularized counts for tackling the heteroskedasticity of data for visualization of gene expression ([25]; statistics Table S1). We selected A375 (*mut*BRAF) and WM1346 (*mut*NRAS) for further analysis, since they showed all features of phenotype-switching. Gene expression profiles of parental cells revealed pronounced modifications 6 h after IZI50 stimulation, but remained largely unaffected in IZI5-conditioned cells as illustrated by volcano plots (Fig. 4A). Data implied apoptosis resistance as well as enhanced proliferation/migration to be less dependent on de novo gene synthesis but rather due to changes in pre-existing signal transduction pathways. Venn analysis revealed 1248 intersecting genes to be differentially expressed upon IZI50 treatment in both of the parental, but not IZI5-conditioned cells (Fig. S2). To identify key regulators of consistently affected pathways, we performed GeneWalk analysis, a tool which employs representation learning on gene ontology (GO) information and allows identifying key genes and their relevant functions [26]. GeneWalk-predicted upstream regulators were plotted according to the ‘Fraction of Relevant GO Terms per Gene’ *versus* ‘Number of GO Annotations per Gene’, and the number of direct interaction partners within the regulatory network indicated by the size of the respective dots. Top regulators that are active and relevant are presented in the upper right corner, followed in priority by regulators plotted in the bottom right corner (Fig. 4B). From these two groups of high priority regulators we selected 18 candidates (Table S2) that interacted with 20 or more partners within the regulatory network. Out of these 18 candidates, 10 (MET, PML, PRKDC, RARA, TRAF6, FAK(PTK2), IQGAP1, ITGAV, FN1, RB1) served a function in the regulation of “cell death/survival”, “migration/invasion”, or “tumorigenesis”. However, only six (MET, PML, PRKDC, FAK(PTK2), IQGAP1, ITGAV) showed a meaningful (genes regulated in the context of metastatic phenotype) and coherent (analogous gene regulation) expression pattern in *mut*BRAF and *mut*NRAS cells. In particular, focal adhesion kinase FAK(PTK2) caught our attention, because it is capable of conferring cell death resistance and to promote proliferation, migration and invasion of cancer cells [15, 17, 27]. FAK gene expression was coherently upregulated in IZI5-conditioned A375 and WM1346 cells, and pronouncedly downregulated in parental cells in response to IZI50 (Fig. 4C; log2FoldChanges/p-values Table S3). Western-blot analysis did not confirm significant upregulation of FAK protein in IZI5-conditioned cells, but revealed that cleavage of full length 125 kDa FAK into a ~90 kDa fragment exclusively occured in parental cells upon IZI50 treatment (Fig. 4D). FAK cleavage was caspase-dependent, because it could be prevented by pan-caspase inhibition using QVD (Fig. 4E). Specifically, FAK processing appeared to be facilitated by caspase-8, because it remained absent in WM115 MM clones #1 and #2 in which caspase-8 had been knocked out by CRISPR-Cas9 technology (Fig. 4F). Thus, reduced caspase-8 expression in IZI5-conditioned cells (Fig. 1B) may contribute to TRAIL resistance and enhanced migration of MM cells by impeding FAK cleavage.

### Integrin-mediated FAK activation causes upregulation of MAPK signaling pathways

**Fig. 5:**
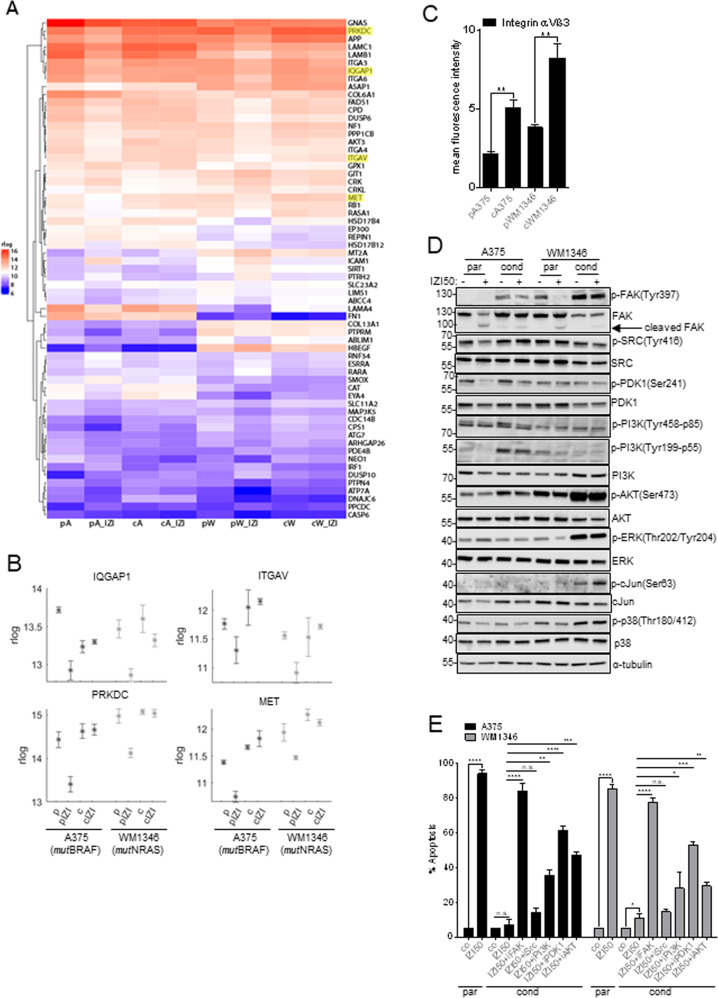
Integrin-mediated FAK activation causes upregulation of MAPK signaling pathways. **A** Mean expression (rlog counts) of the FAK/PTK2 interaction partners in the GeneWalk regulatory network in the different contexts of A375 and WM1346 cells. p=parental; c=conditioned; IZI = stimulated with 50 ng/ml IZI1551; A = A375; W = WM1346. FAK interaction partners that also represented within the 18 top regulators are marked in yellow. **B** Mean expression (rlog counts) of PRKDC, IQGAP, ITGAV, MET genes in parental (p) and IZI5-conditioned (c) A375 and WM1346 cells in response to IZI50 treatment for 6 h. **C** The surface expression of integrin αVβ3 on unstimulated parental (p) and IZI5-conditioned (c) A375 and WM1346 cells was determined by flow cytometry (*n* = 3: **p ≤ 0.01). **D** Parental (p) and IZI5-conditioned (c) A375 and WM1346 cells were stimulated with IZI50. After 24 h the phosphorylation status of FAK, SRC, PDK1, PI3K, AKT, ERK, cJUN, and p38 was monitored by Western-blot analysis with α-tubulin as loading control. One representative out of three independently performed experiments is shown. **E** Parental (p) A375 and WM1346 cells were stimulated with IZI50. IZI-conditioned (c) cells were treated for 1 h with the FAK inhibitor (iFAK; 5 µM) defactinib, the SRC inhibitor (iSrc; 10 µM) dasatinib, the PI3K inhibitor (iPI3K; 20 µM) alpelisib, the PDK1 inhibitor (iPDK1; 10 µM) GSK2334470, and the AKT inhibitor (iAKT; 20 µM) afuresertib, respectively, prior to IZI50 stimulation. After 24 h apoptosis induction was assessed using a CDDE (*n* = 3: *p ≤ 0.05; **p ≤ 0.01; ***p ≤ 0.001; ****p ≤ 0.0001; n.s. = not significant).

Within the GeneWalk regulatory network, FAK was identified to interact with 62 proteins (Fig. 5A), with 41 serving a function in the regulation of “cell death/survival”, “migration/invasion”, or “tumorigenesis” (Table S4). Only 25 of these FAK interactors showed a reasonable and coherent expression pattern in both, *mut*BRAF and *mut*NRAS cells. We identified four candidates (PRKDC, MET, IQGAP1, ITGAV) that directly interacted with FAK and additionally presented as top regulators of the signaling network (Fig. 5A, marked in yellow, Fig. 4B). Expression of the catalytic subunit of DNA-dependent protein kinase (PRKDC), the mesenchymal-epithelial transition factor (MET), and the Ras GTPase-activating-like protein (IQGAP1), respectively, was upregulated in IZI5-conditioned compared to parental cells, and down-regulation of these regulators of DNA repair, migration, invasion, or cell cycle, was attenuated in IZI5-conditioned cells in response to IZI50 (Fig. 5B; log2FoldChanges/p-values Table S5). Elevated expression of integrin αV subunit (ITGAV) in IZI5-conditioned cells was even enhanced upon IZI50 treatment, making ITGAV the most promising candidate for further analysis (Fig. 5B). Integrin αV preferably forms heterodimers with the β3 subunit to facilitate MM cell motility and cancer progression [10]. Consequently, surface expression of the integrin αVβ3 heterodimer was significantly elevated in IZI5-conditioned cells (Fig. 5C), and may therefore promote activation of FAK. Accordingly, initial FAK Tyr397 phosphorylation was markedly enhanced in IZI5-conditioned cells and remained unaffected upon IZI50-mediated TRAIL-receptor activation. Where present in parental cells, basal FAK-phosphorylation disappeared upon IZI50 stimulation, most likely due to FAK cleavage (Fig. 5D). In IZI5-conditioned *mut*BRAF A375 cells FAK activation coincided with PDK1- and PI3K-driven activation of downstream AKT, whereas *mut*NRAS WM1346 cells responded with PDK1- and PI3K-independent AKT activation as well as enhanced ERK-p38-cJun signaling, any of which is known to promote cell survival, proliferation and migration [16] (Fig. 5D). Hence, FAK may act in concert with or upstream of PI3K, PDK1 and SRC activation, to prevent cell death and concomitantly promote proliferation and migration of MM. Accordingly, re-sensitization of IZI5-conditioned A375 and WM1346 cells was shown to be most pronounced upon inhibition of FAK with defactinib (iFAK), compared to individual inhibition of PI3K (alpelisib), PDK1 (GSK2334470), AKT (afuresertib), and SRC (dasatinib) (Fig. 5E), and showed lowest toxicity towards untransformed cells of the skin (Fig. S3). Consequently, inhibition of FAK may provide a reasonable and tumor selective strategy to sensitize MM to cell death, irrespective of their BRAF/NRAS mutation status.

### Nuclear FAK prevents p53 activation in response to IZI50 treatment

**Fig. 6:**
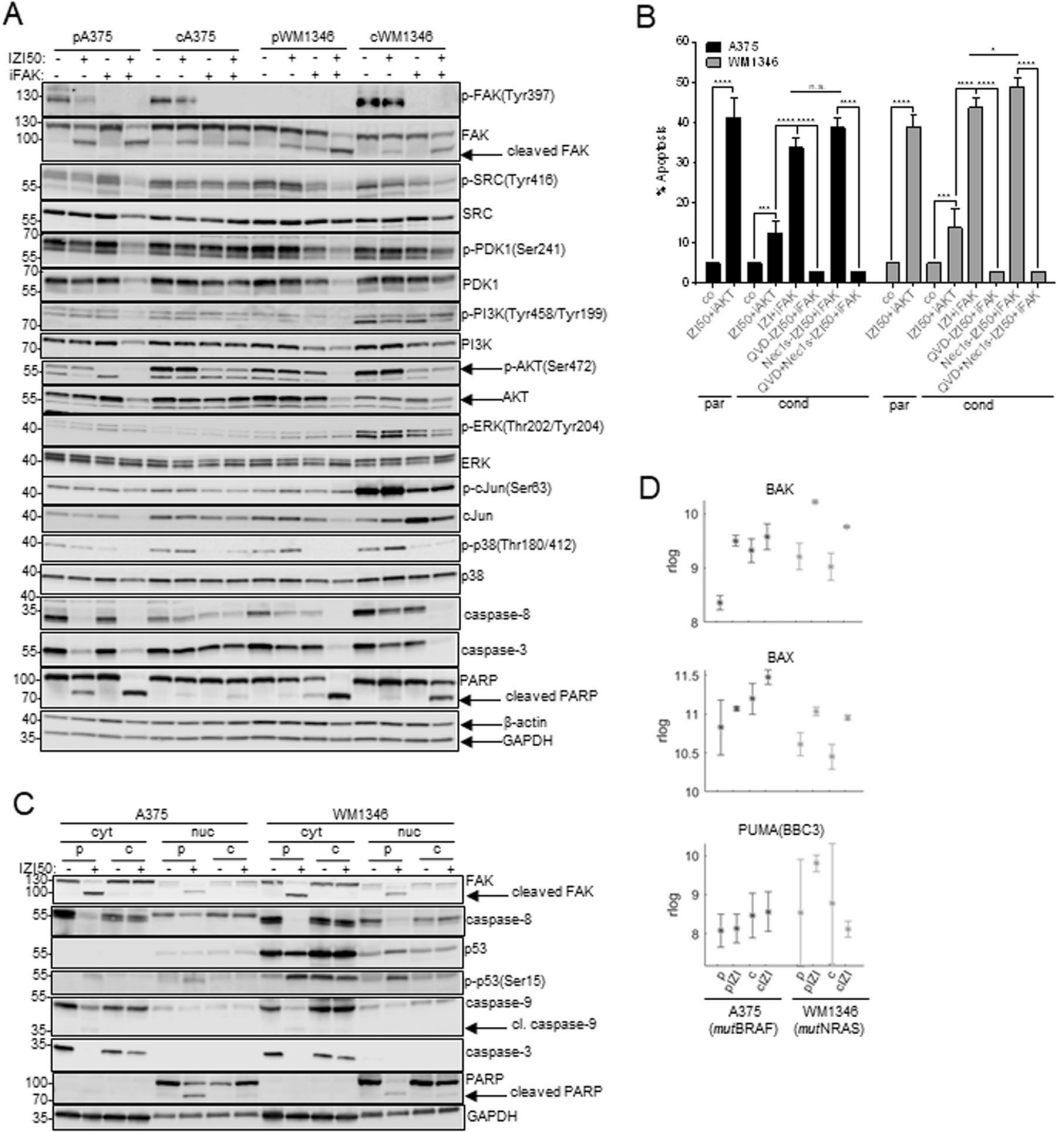
Nuclear FAK prevents p53 activation in response to IZI50 treatment. **A** Parental (p) and IZI5-conditioned (c) A375 and WM1346 cells were stimulated with IZI50, 5 µM defactinib (iFAK), or both. After 24 h the phosphorylation status of FAK, SRC, PDK1, PI3K, AKT, ERK, cJUN, and p38, as well as processing of caspase-3 and PARP was monitored by Western-blot analysis with β-actin and GAPDH as loading controls. One representative out of three independently performed experiments is shown. **B** Parental (p) A375 and WM1346 cells were stimulated with IZI50. IZI5-conditioned (c) cells were treated with either 5 µM QVD, 15 µM Nec1s, or both for 1 h prior to combined FAK inhibitor (iFAK) defactinib and IZI50 stimulation. After 24 h apoptosis induction was assessed using a CDDE (*n* = 3: *p ≤ 0.05; ***p ≤ 0.001; ****p ≤ 0.0001; n.s. = not significant). **C** Parental (p) and IZI5-conditioned (c) A375 and WM1346 cells were stimulated with IZI50. After 24 h the status of FAK, p53, p-p53(Ser15), caspase-8, caspase-3, caspase-9 and PARP, respectively, was determined in cytosolic (cyt) and nuclear (nuc) protein fractions by Western-blot analysis, with GAPDH as loading control. One representative out of three independently performed experiments is shown. **D** Mean expression (rlog counts) of pro-apoptotic BAK, BAX and PUMA genes in parental (p) and IZI5-conditioned (c) A375 and WM1346 cells in response IZI50 treatment for 6 h.

To attribute a dual role in phenotype-switching of IZI5-conditioned MM cells to FAK, we investigated the impact of FAK inhibition on both, cell survival and cell death. Defactinib fully abolished phosphorylation of FAK and consequently of PI3K, PDK1, AKT, ERK, p38, cJun, and SRC where applicable, and resulted in processing of caspase-3 and cleavage of PARP (Fig. 6A). In concert with caspase activation, cleavage of FAK occurred, thereby presumably promoting apoptosis in a positive feedback mechanism. Accordingly, re-sensitization of IZI5-conditioned A375 and WM1346 cells to IZI50 by FAK inhibition was completely prevented by the pan-caspase inhibitor QVD, but not by the RIP1-inhibitor Nec1S (Fig. 6B).

Besides its function as a cytosolic tyrosine kinase, nuclear FAK is able to prevent *wt*p53 accumulation and consequently tumor cell death independent of its kinase function [19]. Hence, a fraction of FAK and caspase-8, but not caspase-3 or caspase-9, localized to the nuclear compartment of both, parental and IZI5-conditioned MM cells (Fig. 6C). In IZI50-stimulated parental cells, activation of caspase-8 and consequently cleavage of nuclear FAK nicely coincided with an accumulation and activation of p53, being evident by Ser15 phosphorylation. (Fig. 6C). Consequently, upregulation of pro-apoptotic BAK, BAX and PUMA genes (Fig. 6D, log2FoldChanges/p-values Table S6) and intrinsic apoptosis becomes initiated in a caspase-9/caspase-3-dependent manner [28] (Fig. 6C). In IZI5-conditioned cells, integrin-mediated activation of cytosolic FAK triggers proliferation and migration in a MAPK-dependent manner, whereas nuclear FAK prevents *wt*p53-induced apoptosis induction. Upon TRAIL-receptor activation in parental cells, proteolytic activation of caspase-8 causes cleavage of FAK in the cytosol and the nucleus thereby counteracting both functions of FAK, and consequently executing apoptosis.

### TRAIL-resistance and tumor outgrowth of IZI5-conditioned MM cells depends on FAK

**Fig. 7:**
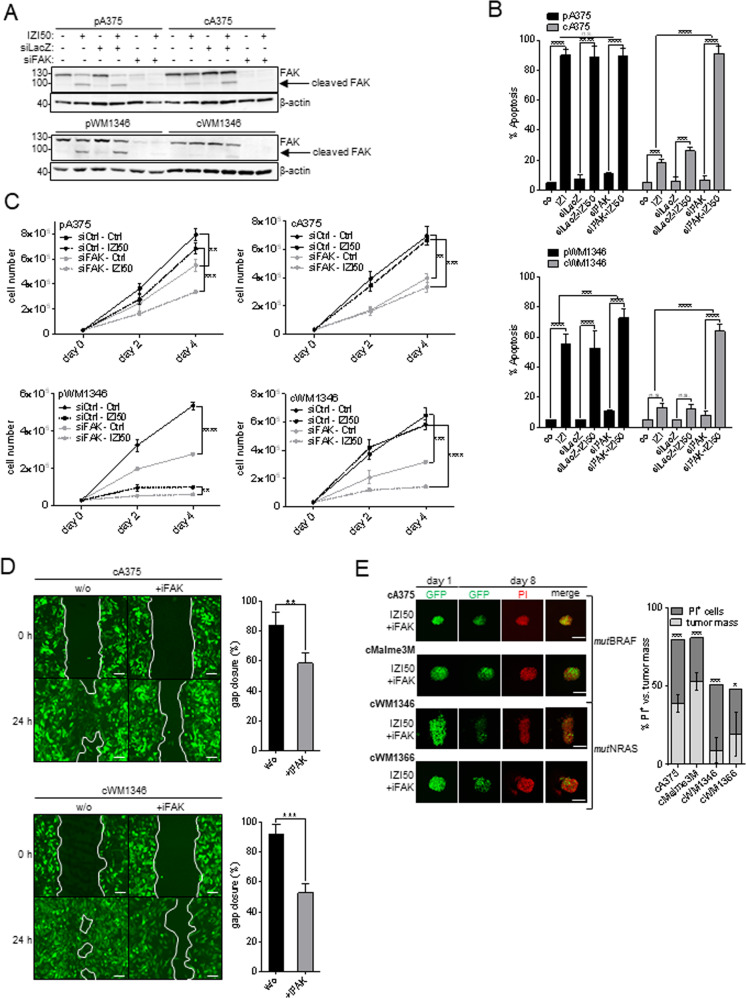
TRAIL-resistance and tumor outgrowth of IZI5-conditioned MM cells depends on FAK. **A** FAK was depleted in parental (p) and IZI5-conditioned (c) A375 and WM1346 cells using RNA interference (siFAK), with siLacZ as scrambled control. Transient knockdown of FAK was monitored 24 h later by Western-blot analysis, with β-actin as loading control. One representative out of three independently performed experiments is shown. **B** Cells as in **A** were stimulated with IZI50 and apoptosis induction determined 24 h later using a CDDE (*n* = 3: ***p ≤ 0.001; ****p ≤ 0.0001; n.s. = not significant). **C** Unstimulated cells as in **A** were subjected to a proliferation assay and cell numbers quantified at the indicated time points (*n* = 3: **p ≤ 0.01; ***p ≤ 0.001; ****p ≤ 0.0001). **D** GFP-expressing IZI5-conditioned (c) A375 and WM1346 cells were left untreated or treated with the FAK inhibitor defactinib (iFAK, 5 µM). Migration of cells was monitored by scratch assay (scale bar = 100 µm) and gap closure quantified after 24 h (*n* = 3: **p ≤ 0.01; ***p ≤ 0.001). **E** GFP-expressing spheroids consisting of IZI5-conditioned (c) *mut*BRAF (A375, Malme3M) and *mut*NRAS (WM1346, WM1366) melanoma cells, respectively, were individually embedded into 3D dextran-based gel-matrices and stimulated with IZI50 and 5 µM defactinib (iFAK) every other day over 8 days (scale bar = 200 µm). At day eight cell death was visualized by addition of 6.7 µg/ml PI. Confocal images of individual spheroids were taken at day one and eight and the tumor volume (green) *versus* tumor death (red) quantified at day eight (*n* = 3: *p ≤ 0.05; ***p ≤ 0.001).

To consolidate that phenotype-switching of IZI5-conditioned MM cells specifically depended on FAK, we knocked down FAK in A375 and WM1346 cells by RNA interference (Fig. 7A). FAK depletion fully re-sensitized IZI5-conditioned cells to IZI50-induced apoptosis (Fig. 7B), and significantly reduced proliferation (Fig. 7C). Pharmacological inhibition of FAK with defactinib, significantly reduced migration of IZI5-conditioned cells (Fig. 7D), almost back to the level of parental cells (compare Fig. 3B), and completely abolished outgrowth of cells from as well as increase in the volume of 3D spheroids consisting of any of the four IZI5-conditioned MM cells (*mut*BRAF A375, Malme3M; *mut*NRAS WM1346, WM1366) in response to IZI50 treatment. Instead, apoptosis was induced as determined by PI incorporation (Fig. 7E, compare Fig. 1B), allowing to conclude that FAK inhibition may universally break TRAIL resistance and prevent invasiveness of MM independent of the intrinsic mutation status.

### FAK inhibition sensitizes resistant *mut*BRAF and *mut*NRAS melanoma to mutation-specific targeted kinase inhibition

**Fig. 8:**
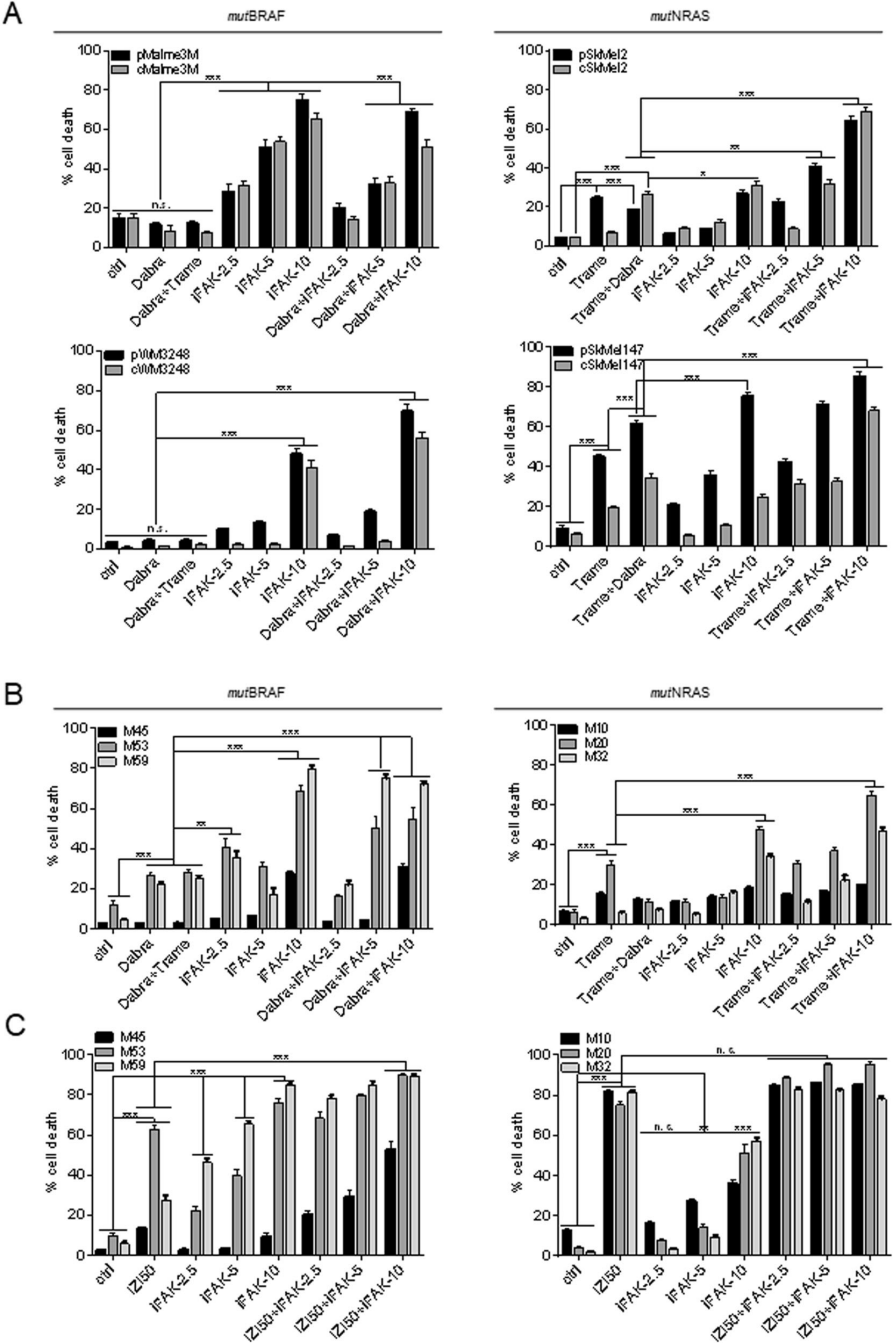
FAK inhibition sensitizes resistant *mut*BRAF and *mut*NRAS melanoma to mutation-specific targeted kinase inhibition. **A** Parental *mut*BRAF Malme3M and WM3248 MM cells were conditioned to 1 µM BRAF inhibitor dabrafenib (Dabra); *mut*NRAS SkMel2 and SkMel147 cells to 1 nM MEK inhibitor trametinib (Trame). *mut*BRAF parental (p) and conditioned (c) cells were treated with 10 µM dabrafenib, dabrafenib + 1 µM trametinib, 2.5, 5, or 10 µM of the iFAK defactinib alone and in combination with 10 µM dabrafenib. Parental (p) and conditioned (c) *mut*NRAS cells were treated with 1 µM trametinib, trametinib + 10 µM dabrafenib, 2.5, 5 or 10 µM of the iFAK defactinib alone and in combination with trametinib. Cell death was monitored by PI (1 µg/ml) uptake using IncuCyte^®^ live-cell analysis for 48 h. **B** Three *mut*BRAF (M45, M53, M59) and three *mut*NRAS (M10, M20, M32) cell samples freshly isolated from patients with relapsed melanoma metastases were treated as in **A** according to their mutation status. Cell death was monitored by PI (1 µg/ml) uptake using IncuCyte^®^ technology for 48 h. **C** The same patient-derived metastatic cell samples as in (**B**) were treated with IZI50, 2.5, 5, or 10 µM of the iFAK defactinib alone and in combination with IZI50. Cell death was monitored by PI (1 µg/ml) uptake using IncuCyte^®^ technology for 48 h. For each experiment *n* = 3 is shown (*p ≤ 0.05; **p ≤ 0.01; ***p ≤ 0.001; n.s. = not significant).

Since cytosolic FAK acts upstream of BRAF/NRAS, and nuclear FAK interferes with the activation of *wt*p53 as being expressed in the majority of melanomas, we investigated whether FAK inhibition may have an implication in current clinical applications, i.e. re-sensitizing MM cells that have overcome mutation-specific targeted kinase inhibition to cell death. To mimic the relapse situation we conditioned *mut*BRAF Malme3M and WM3248 cells to 1 µM dabrafenib and *mut*NRAS SkMel2 and SkMel147 cells to 1 nM trametinib for 6 months. Subsequently, parental and conditioned cell pairs were treated with the respective mutation-specific kinase inhibitors alone or with the clinically relevant combination of *mut*BRAF/MEK inhibitors (10 µM dabrafenib + 10 nM trametinib). In parallel, we applied three different doses of the FAK inhibitor defactinib (2.5, 5, 10 µM) alone or combined with the mutation-specific kinase inhibitor (Fig. 8A). In all cell lines, cell death induction by 10 µM defactinib alone was significantly superior to individual or combined *mut*BRAF/MEK inhibition, as determined by PI uptake (Fig. 8A). Especially in *mut*BRAF cells about 60–80% of cell death was achieved compared to only 10–20% upon dabrafenib/trametinib application. These differences were explicitly lower in *mut*NRAS cells, nevertheless, combined inhibition of FAK and the individual mutation-specific kinases yielded strongest cell death in all cell lines and caused re-sensitization of conditioned cells irrespective of the intrinsic mutation status (Fig. 8A). Strikingly, FAK inhibition also re-sensitized therapy-resistant MM cells, isolated from three *mut*BRAF (M45, M53, M59) and three *mut*NRAS (M10, M20, M32) metastasis relapsed from targeted kinase-inhibition. As expected, melanoma samples only poorly responded to treatment with individual or combined mutation-specific kinase inhibitors (Fig. 8B). Exposure to 10 µM FAK inhibitor defactinib alone or combined with either dabrafenib or trametinib—depending on the mutation status—yielded 70–80% cell death in two *mut*BRAF (M53, M54) and two *mut*NRAS (M20, M32) melanoma samples, and improved cell death induction in M45 and M10 cells compared to the clinically relevant drug combination (Fig. 8B). Primary melanocytes, keratinocytes, and fibroblasts remained largely unaffected by the most potent drug combinations, accounting for their tumor selectivity (Fig. S4). Finally, TRAIL-receptor activation using IZI50 was even superior in subjecting all metastatic cell samples to cell death, irrespective of the mutation status, and reached almost 100% cell death of targeted kinase inhibitor-resistant *mut*NRAS MM cells. Taken together, we have identified FAK as a potential target to improve second-line therapy for relapsed MM patients, especially when combined to second generation TRAIL-receptor agonists, like IZI1551.

## Discussion

To date, clinical intervention strategies for MM are exclusively based on the mutation status of key drivers of MM progression, BRAF and NRAS oncogenes. However, other pathophysiological modifications may contribute to therapy resistance and tumor relapse, including tumor cell specific activation of alternative kinases within the signal transduction network [29–32].

Accordingly, high unmet medical need still exists for new treatment options in particular for patients that have acquired resistance to conventional treatment regimens. In this context, 2^nd^ generation TRAIL-receptor agonists with enhanced bioactivity, may overcome the limitations of trimeric TRAIL molecules and receptor-agonistic antibodies that have previously failed in clinical trials [33]. We previously showed hexameric TRAIL-receptor-agonist IZI1551 (IZI) [20, 21] to be superior in inducing apoptotic cell death in *mut*BRAF/MEK inhibition-sensitive and -resistant MM cells [22]. However, long-term exposure to the EC_50_ dose IZI5, as it may occur within heterogeneous tumor populations, changed the responsiveness to lethal IZI50, causing acquired resistance and a pronounced metastatic phenotype, involving enhanced proliferation, migration, clonogenic outgrowth, and increased tumor volume. A similar phenotype-switch is also observed upon acquired resistance of MM against targeted kinase inhibitors in the clinic and hinders second-line treatment regimens [34, 35].

Performing gene ontology analysis with GenWalk we identified FAK as a key player in conferring both apoptosis resistance and an enhanced metastatic phenotype in IZI5-conditioned *mut*BRAF and *mut*NRAS MM cells. In concert with integrin-mediated signaling a function in invasion and metastatic characteristics of the epithelial-mesenchymal transition has been attributed to FAK [13, 15]. Likewise, increased FAK expression has been implemented in survival of other cancer types, including breast cancer [36, 37], bladder cancer [38] and squamous cell carcinoma [39] through activation of MAPK- and PI3K-related signaling pathways [40]. Following this line, we show that MM cells that had acquired resistance to IZI1551, presented with increased integrin αVβ3 expression and consequently constitutive FAK activation, followed by downstream activation of MAPK and PI3K-driven survival pathways. In contrast, FAK was found to be largely de-phosphorylated and proteolytically cleaved by caspase-8 in IZI50-stimulated parental cells, thereby preventing the metastatic phenotype and inducing apoptosis. Cleavage of both, caspase-3 and FAK has previously been reported to occur in cisplatin-sensitive ovarian cancer cells, and could be prevented upon application of the caspase-3 inhibitor zDEVD [41]. Along this line, sensitization of irradiation-resistant glioma cells with temozolomide antagonized integrin αVβ3 expression and FAK phosphorylation, causing caspase-3-mediated FAK cleavage and cell death induction [42]. Early studies suggested caspase-mediated cleavage of FAK to contribute to TRAIL-induced cell death [43]. Vice versa, FAK was shown to inhibit death receptor-induced apoptosis through binding to receptor-interacting protein (RIP) [44]. In all these studies it remained, however, unsolved whether FAK cleavage was a cause or a consequence of apoptosis induction. In this context, de-phosphorylation of FAK was shown to precede its cleavage by caspases in renal epithelial cells [45]. Even though it cannot be fully excluded that caspase-3 may also cleave FAK in the process of TRAIL-induced cell death, we here show that cytosolic caspase-8-mediated cleavage of FAK is essential for full apoptosis induction in response to TRAIL-receptor activation, because depletion of caspase-8 fully prevented processing of FAK in MM cells that still expressed downstream caspases. Additionally, caspase-8-mediated cleavage of FAK also takes place in the nucleus, in the absence of caspase-3. This is of particular interest, because we could recently show caspase-8 to be expressed in the nucleus of MM and other cancer types, where it employs destabilization of *wt*p53 and consequently therapy resistance through cleavage of USP28 [46]. Cleavage of FAK resulted in *wt*p53 activation and induction of intrinsic apoptosis, implying that the FERM domain of cleaved FAK can no longer serve as a scaffold for *wt*p53/MDM2 interaction. Hence, enhanced FAK-mediated *wt*p53 turnover represents another important mechanism how MM cells, may resist therapeutic intervention [47].

Taken together, we present how FAK may contribute to treatment resistance and a metastatic phenotype in *wt*p53-expressing MM. Cytosolic FAK triggers survival pathways in a PI3K- and MAPK-dependent manner, whereas concomitantly nuclear FAK prevents *wt*p53 activation. FAK activation can be counteracted through cleavage by caspase-8 to antagonize both pro-cancerous actions. FAK inhibition reverted the metastatic phenotype-switch evoked by IZI5-conditioning, and restored IZI responsiveness irrespective of the BRAF/NRAS mutation status. FAK inhibition also re-sensitized kinase inhibitor-resistant *mut*BRAF and *mut*NRAS MM cell lines as well as patient samples that had relapsed from targeted kinase inhibition. Based on our data FAK-inhibition alone or in combination with 2^nd^ generation TRAIL-receptor agonists may be recommended for treatment of primary or therapy resistant relapsed MM metastasis irrespective of the intrinsic mutation status.

## Materials and methods

### Cells and reagents

Human melanoma cell lines (A375, Malme3M, Skmel2, Skmel147, WM115 WM1366, WM1346) were obtained from the American Type Culture Collection (ATCC) and maintained in RPMI 1640 medium (Invitrogen, Karlsruhe, Germany) with 10% FCS in a humified atmosphere of 5% CO_2_ at 37 °C. Primary melanoma cells were freshly isolated from patients metastasis (M10 = m (58); M20 = m (87); M32 = m (53); M45 = m (41); M53 = m (73); M59 = m (48)) by incubating the chopped tissue samples in HBBS (w/o Ca^2+^ and Mg^2+^) containing 0.05% collagenase, 0.1% hyaluronidase, 1.25 U/ml dispase, 20 mM HEPES, 100 g/ml gentamycin, 100 U/ml penicillin and 100 g/ml streptomycin for 60 min at 37 °C. After centrifugation cell pellets were washed in HBSS/20 mM HEPES and maintained in RPMI + 10% FCS. The usage of patient material (metastasis) for biochemical analysis was approved by the ethics committee of the TU-Dresden (EK 335082018) and informed consent was obtained from all patients. Primary cells were purchased from Cell Systems (Troisdorf, Germany) and used at passage 4. Keratinocytes were maintained in Keralife medium (Cell Systems), fibroblasts in DMEM (Invitrogen), and melanocytes in Melanocyte Growth Medium (M2, Promocell, Heidelberg, Germany). All cell lines were tested every other month to be mycoplasma-negative as judged by the MocyAlert Mycoplasma Detection Kit (LT-07, Lonza, Basel, Switzerland). Cell conditioning was achieved by long-term exposure of melanoma cells to different compounds over a period of 6 months. A375, Malme3M, WM1366, and WM1346 cell lines were conditioned to 5 ng/ml of the hexavalent TRAIL-receptor-agonist IZI1551 generated at the Institute of Cell Biology and Immunology, University of Stuttgart [20, 21]. WM3248 and Malme3M cell lines were conditioned to 1 µM of the *mut*BRAF inhibitor dabrafenib (#S2807; Selleckchem, Munich, Germany) and SkMel2 and SkMel147 cells to 1 nM of the MEK inhibitor trametinib (#S2673; Selleckchem), respectively.

For stimulation of cells, the pan-caspase inhibitor QVD (Novus Biologicals, Littleton, CO, USA) was added at 5 µM, the RIP1-inhibitor Nec1S (BioVision, Hannover, Germany) at 15 µM, the FAK inhibitor defactinib (#S7654; Selleckchem) at 2.5–10 µM, the SRC inhibtitor dasatinib (#S1021; Sellekchem) at 10 µM, the PDK1 inhibitor GSK2334470 (#S7087, Sellckchem) at 20 µM, the PI3K inhibitor alpelisip (#HY-15244; MedChemExpress, Moumouth Junction, NJ, USA) at 20 µM, the AKT inhibitor afuresertib (#S7521; Selleckchem) at 10 µM, dabrafenib at 10 µM, and trametinib at 10 nM.

### 3D melanoma spheroids

Melanoma spheroids were generated using the “hanging drop” method [24]. Briefly, (4–20) × 10^4^ GFP-expressing melanoma cells were resuspended in 5 ml of RPMI containing 20–40% methocell, depending on cell line. 40 drops of 25 µl containing 200–1000 cells were spotted on the lid of a 10 cm cell culture dish, inverted onto the dish filled with 10 ml of 1x PBS, and incubated for 12 days at 37 °C, 5% CO_2_.

For analysis, mature spheroids were embedded into 3D dextran-based gel-matrices containing 6 nMol/L of thiol-reactive groups and thiol groups (3-D Life Dextran-CD Hydrogel SG, #G93–1; Cellendes, Reutlingen, Germany), according to the manufacturer’s protocol. Dextran was used as the thiol-reactive polymer and crosslinked with a polyethylene glycol peptide conjugate (CD-Link), which contains a matrix-metalloprotease cleavable peptide that allows cells to cleave the crosslinker in order to spread and migrate throughout the gel. One single spheroid was injected per 30 µl gel-matrix in a volume of 5 µl medium and incubated for 30 min at 37 °C. Subsequently, gels were covered with medium and drugs added every other day. Development of individual spheroids was monitored daily by confocal fluorescence microscopy (LSM 780/FCS inverse, Zeiss, Germany) equipped with a Plan-Apochromat ×10/0.45 M27 objective, over 8 days. At day eight cell death of spheroids was visualized by the addition of 6.7 µg/ml propidium iodide (PI, Invitrogen) in PBS for 20 min at RT in the dark. After washing twice with PBS confocal images were taken. For the emitted green fluorescent, the laser emission peak was 488 nm (emission filter 499–597 nm). For the emitted red fluorescent, the laser emission peak was 561 nm (emission filter 606–686 nm).

### Proliferation and clonogenic outgrowth

(8–10) × 10^4^ cells were seeded in 6-well plates and incubated with or without the indicated drug for 48 h. Subsequently, cells were harvested, counted and re-seeded at the starting density every other day for three times. The progressive increase of cells was calculated as proliferation rate over time.

Clonogenic outgrowth was determined by seeding (1–6.5) × 10^3^ cells into 6-well plates for 8 days or until control cells had reached confluency. Subsequently, cells were stained with crystal violet (0.1 w/v in 20% methanol) for 15 min at RT. After washing with PBS, crystal violet was dissolved from cells with 0.1 M KH_2_PO_4_/EtOH for 5 min at RT and color intensity of supernatants measured at 595 nm (Tecan M200, Tecan, Maennedorf, Switzerland).

### Scratch assay

6.5 × 10^5^ GFP-expressing cells were plated onto cell culture dishes (35 × 10 mm) at 90% confluency. After 24 h, a 1-mm wide scratch was inserted across the cell layer using a sterile (10 µl) pipette tip. Closure of the scratch by cell migration was monitored over 48 h using smart fluorescent cell analyser (JuLI^TM^_,_ VWR International GmbH, Germany).

### Determination of cell death

Apoptosis was determined in a Cell Death Detection ELISA (CDDE, Roche, Mannheim, FRG). The enrichment of mono- and oligonucleosomes released into the cytosol is calculated: absorbance of samples/absorbance of control cells (Tecan M200). An enrichment factor of 2 corresponds to 10% apoptosis as determined by AnnexinV FACS analysis (FACSAria III, Becton Dickinson, Heidelberg, Germany).

Sensitivity towards kinase inhibitors was monitored by PI (1 µg/ml) uptake using automated, image-based IncuCyte^®^ (Satorius, Goettingen, Germany) screening technology over a period of 48 h, taking images every 2 h.

### Plasmid and siRNA transfection

For stable expression of GFP, 6.5 × 10^6^ A375, Malme3M, WM1346 and WM1366 cells were electroporated with 25 µg of pEGFP plasmid in 600 µl medium containing 2% DMSO and maintained in selection medium containing 1 mg/ml Geneticin G418 Sulfate (sc-29065; Santa Cruz Biotechnology Inc, Santa Cruz, CA, USA). GFP-positive cells were enriched by cell sorting (FACSAria III). For CRISPR/Cas9-mediated knock out of caspase-8 WM115 melanoma cells were transfected with pSpCas9(BB)-2a-GFP (PX458)-hCASP8 gRNA2.2 (clone #1), and pSpCas9(BB)-2a-GFP (PX458)-hCASP8 gRNA3.1 (clone #2), respectively, and GFP-positive cells were enriched by cell sorting (FACS Aria III). Plasmids were kindly provided by Prof. Dr. Hamid Kashkar, Institute for Medical Microbiology, Immunology and Hygiene, University of Cologne, Germany.

Transient knockdown was facilitated by transfecting 5 × 10^4^ cells with 40 pmol of the respective siRNA for FAK (#6472, Cell Signaling, Danvers, MS, USA), and lacZ-5′-GCGGCUGCCGGAAUUUACCTT-3′ (MWG Eurofins, Ebersberg, Germany) using Lipofectamine RNAiMax (Invitrogen) 24 h prior to stimulation.

### Western-blot analysis

Cells were lysed in lysis buffer (50 mM HEPES, pH 7.5; 150 mM NaCl; 10% glycerol; 1% Triton-X-100; 1.5 mM MgCl_2_; 1 mM EGTA; 100 mM NaF; 10 mM pyrophosphate, 0.01 % NaN_3_, phosSTOP^®^ and Complete^®^). For sub-cellular fractionation NE-PER™ Nuclear and Cytoplasmic Extraction Reagents (Thermo Scientific, Waltham, MS, USA) were used according to the manufacturer’s instructions. After centrifugation, supernatants were collected and the protein content determined by DC Protein assay kit (BioRad, Hercules, CA, USA). 60–80 µg of protein extracts were subjected to 4–15% gradient SDS-PAGE (BioRad), blotted onto nitrocellulose membranes using the Trans-Blot^®^ Turbo™ Transfer System (BioRad), blocked in 1% blocking solution (Roche) and incubated with antibodies directed against PARP, p53 (BD-Biosciences; #551025, #554293), caspase-8 (#AG-20B-0057; Adipogen, San Diego, CA, USA), caspase-3, caspase-9, AKT, p-AKT(S473), ERK1/2, p-ERK1/2(T202/Y204), FAK, p-FAK(Y397), cJun, p-cJun, PDK1, p-PDK1(S241), PI3K, p-PI3K(Y458-p85/Y199-p55), p38, p-p38(T180/412), p-p53(S15), SRC, p-SRC(Y416), (Cell Signaling; #9665, #9502, #2920, #4060, #9102, #4376, #3285, #8556, #9165, #9261, #3062, #3061, #4292, #17366, #9212, #9216, #9248, #2108, #6943), respectively, diluted in 0.5% blocking solution. Equal loading was monitored by re-probing membranes with antibodies against β-actin, GADPH (Cell Signaling: #4970, #2118), or α-tubulin (Thermo Scientific: #MS-581-P1). HRP-conjugated secondary antibodies were purchased from GE-Healthcare (Anti-mouse-HRP, #NA931; Anti-rabbit-HRP, #NA934). Bands were visualized by applying chemiluminescence SuperSignal^®^ detection systems (Thermo Scientific).

### Flow cytometry analysis

5 × 10^5^ cells were blocked in PBS/2% BSA for 30 min and incubated with a fluorescently labeled anti αVβ3 integrin antibody (Alexa Fluor^®^ 488 anti-human CD51/61, #304408, Biolegend, San Diego, CA, USA) at 1 mg/ml in PBS/2% BSA, for 1 h on ice. After washing cells were subjected to FACS analysis (FACSAria III). Excitation wavelength used was 488 nm. The emitted green fluorescence (lmax 520 nm) was detected using (FL1) band-pass filter.

### RNA extraction and RNAseq analysis

RNA was extracted from 5 × 10^6^ cells using RNeasy Mini-Kit (Qiagen, Minden, Germany) according to the manufacturer’s protocol.

Purity of RNA was assessed by determining the ratio of absorbance at 260 nm and 280 nm (M200 Tecan). RNA integrity was investigated with Agilent 2100 Bioanalyzer (Agilent Technologies, Palo Alto, CA, USA). RNA sequencing was performed according to the previously published protocols [48]. Briefly, 1 µg of total RNA was used for the library preparation using TruSeq stranded mRNA library preparation kit (Illumina, San Diego, CA, USA). The libraries were quantified using Qubit dsDNA HS assay kit (ThermoFisher Scientific, Waltham, MA USA) and size distribution was determined using Agilent 2100 Bioanalyzer. All prepared samples were pooled in equimolar concentration and sequenced at LCSB sequencing platform using NextSeq500 (Illumina, San Diego, CA, USA). Reads handling from trimming to feature counting was performed using the workflow manager Snakemake (v5.20.1 [49]) and our RNA-seq template (release v0.2 freely available at https://git-r3lab.uni.lu/aurelien.ginolhac/snakemake-rna-seq). Briefly, the trimming for adapter sequences was done using AdapterRemoval (v2.3.1 [50]) using the following settings: minimal length to retained reads set to 35 bp and adapter 1 set to AGATCGGAAGAGCACACGTCTGAACTCCAGTCAC. Reads were aligned to the human reference genome GRCh38 with the ensembl gene annotation version 100 using STAR (v2.7.4a [51]). Read count generation was performed using featureCounts from RSubread (v2.2.2 [52]) ignoring multimapping reads. Differential expression analysis was performed using raw counts and the DESeq2 package (v1.28.1 [25]. Log_2_-fold change (log_2_FC) shrinkage was performed using apeglm to preserve large effects of true positives (v1.10.0 [53]). Plots were created using ggplot2 (v3.3.2 Wickham 2016), ggforce (v0.3.3 Pedersen 2020, https://cran.r-project.org/web/packages/ggforce/index.html) and ComplexHeatmap (v2.7.8.1 [54]). All config files, code and packages references are available at the following address: https://github.com/sysbiolux/TRAIL_IZI_Melanoma. RNAseq data are available in EBI’s ArrayExpress under accession number E-MTAB-10669 (reviewer link: http://www.ebi.ac.uk/arrayexpress/help/how_to_search_private_data.html; username: Reviewer_E-MTAB-10669; password: 0rqyKI1b).

### Quantification and statistical analysis

Unless stated otherwise, results of Cell Death Detection ELISA and flow cytometry analysis are presented as mean ± SD of three independently performed experiments. Western-blot analysis represents one out of three independently performed experiments. Statistical analysis was performed with unpaired Student’s *t test* using GraphPad PRISM 6 software (https://www.graphpad.com).

Quantification of 3D melanoma spheroids (tumor mass) was performed calculating the area of the green fluorescent spheroid and the red PI stained cells. For the migration and invasion analysis, the total area (in pixels) of the cells was measured, whereas for the scratch assay the size of the gap was determined.

All the quantification measurements of spheroids, migration, scratch, and invasion assays were performed by using Fiji software (https://fiji.sc).

## Supplementary information

Supplemental Material

## Data Availability

RNAseq data are available in EBI’s ArrayExpress under accession number E-MTAB-10669 (reviewer link: http://www.ebi.ac.uk/arrayexpress/help/how_to_search_private_data.html; username: Reviewer_E-MTAB-10669; password: 0rqyKI1b).
