# Supplementary material for: Focal adhesion kinase plays a dual role in TRAIL resistance and metastatic outgrowth of malignant melanoma
Source: Cell Death Dis. 2022 Jan 12;13(1):54. doi: 10.1038/s41419-022-04502-8 (PMC8755828; doi:10.1038/s41419-022-04502-8)
Supplement: Supplementary file 1 — Supplemental Material [file 41419_2022_4502_MOESM1_ESM.docx]

**
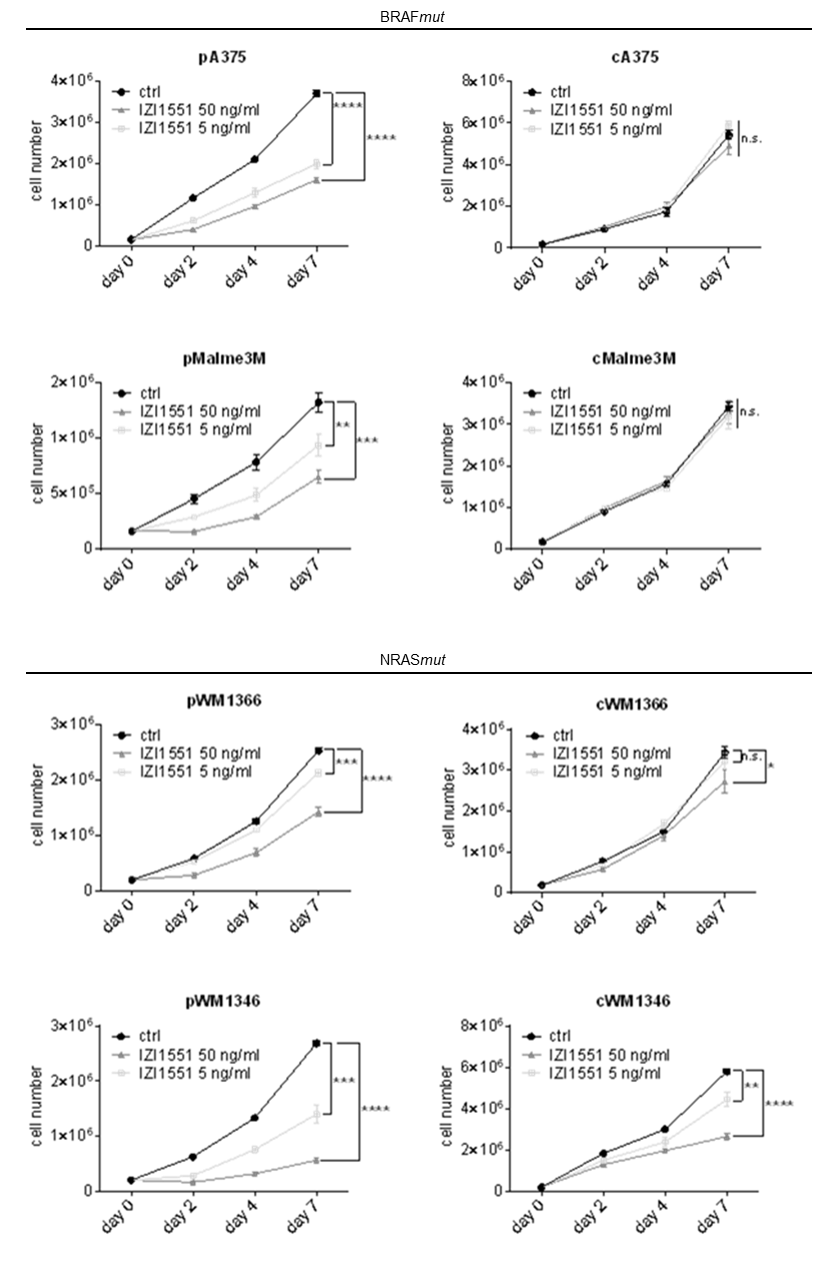
**

**Figure S1. Related to Figure 2. Conditioning to IZI5 enhances proliferation of *mut*BRAF and *mut*NRAS MM cells.**

Parental *mut*BRAF) A375 and Malme3M cells, and *mut*NRA WM1346 and 1366 melanoma cells were conditioned to 5 ng/ml TRAIL receptor agonist IZI1551 (IZI). Parental (p) and IZI-conditioned (c) cells were left untreated or stimulated with 5 and 50 ng/ml IZI1551 (IZI), respectively, every other day. Proliferation of cells in response to IZI1551 was quantified at the indicated time points (n=3: *p ≤ 0.05; **p ≤ 0.01; ***p ≤ 0.001; ****p ≤ 0.0001; n.s. = not significant).


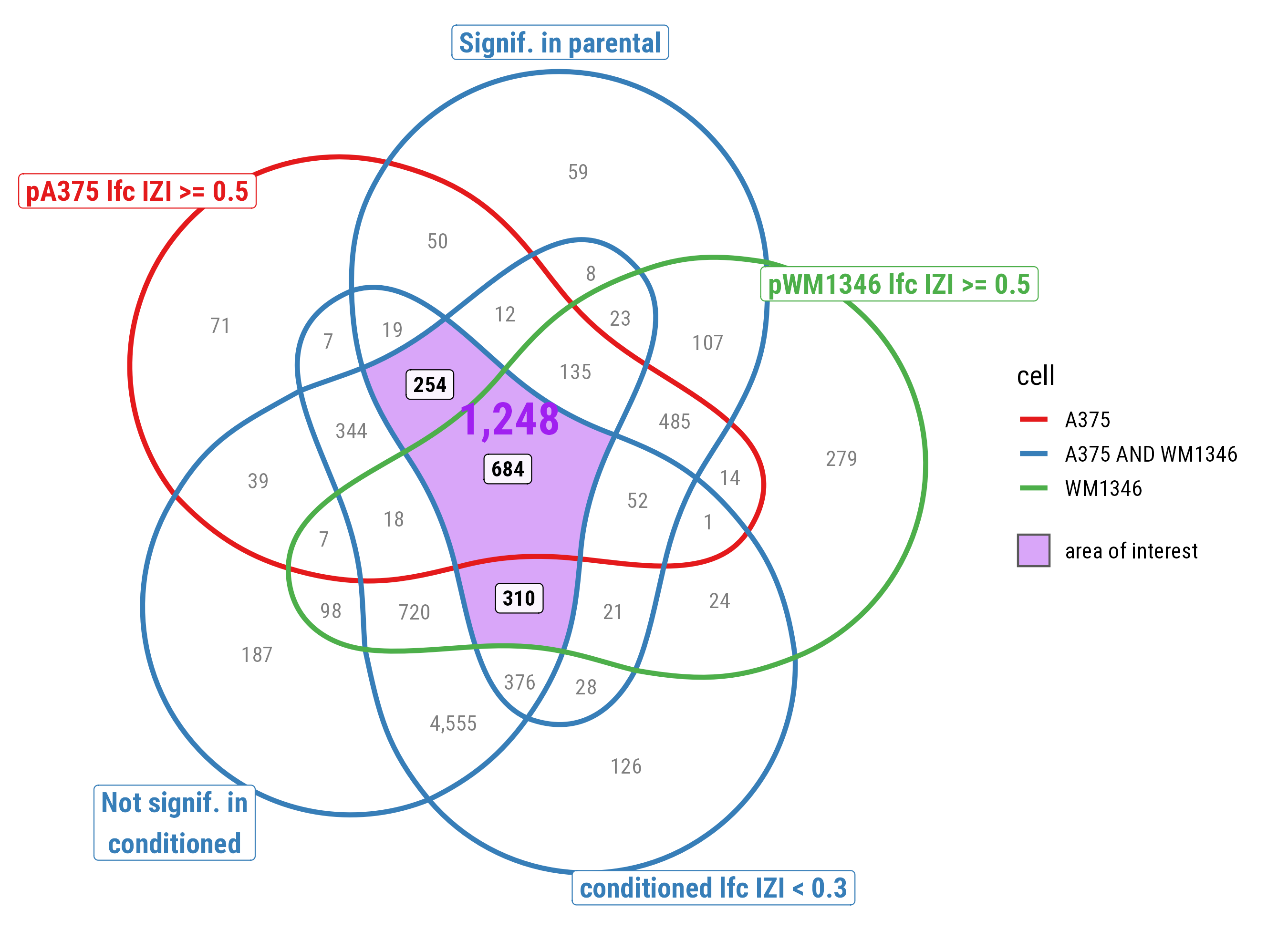


**Figure S2. Related to Figure 4. Selection of coherently expressed genes upon IZI treatment in parental but not conditioned cells**

Among the genes expressed under all eight conditions in both A375 and WM1346 cells(normalized count > 100), the union of the following criteria on adjusted p-values were used:

- Not significant in conditioned: IZI vs control had an adjusted p-value >= 0.05

- Significant in parental: IZI vs control had an adjusted p-value < 0.05

And on log2 fold changes (lfc):

- conditioned lfc IZI < 0.3.

For the final selection, genes with also either a lfc >= 0.5 in A375 parental cells IZI vs control (set in red) or in WM1346 parental cells IZI vs control (set in green) were kept. 684 genes fit all 5 requirements, 310 are within 4 sets but not expressed with a lfc >= 0.5 in pA375 IZI vs control. Reciprocally, 254 are not found expressed with a lfc >= 0.5 in WM1346. In sum of 254, 310 and 684 add up to the 1,248 genes for GeneWalk analysis.

**Figure S3. Related to Figure 5. Primary cells of the skin remain unaffected by FAK inhibition.**

Primary melanocytes, keratinocytes and fibroblasts were treated for 1 h with the FAK inhibitor (iFAK; 5 µM) defactinib, the Src inhibitor (iSrc; 10 µM) Dasatinib, the PI3K inhibitor (iPI3K; 20 µM) alpelisib, the PDK1 inhibitor (iPDK1; 10 µM) GSK2334470, and the AKT inhibitor (iAKT; 20 µM) afuresertib, respectively. After 24 h apoptosis induction was assessed using a CDDE (n=3: ***p ≤ 0.001).

**Figure S4. Related to Figure 8. Primary cells of the skin remain unaffected by combined Dabrafenib/Trametinib treatment and FAK inhibition.**

Primary melanocytes, keratinocytes, and fibroblasts were treated 10 µM dabrafenib, 1 µM trametinib or both, or either dabrafenib or trametinib combined with 10 µM iFAK defactinib. Cell death was monitored by PI (1 µg/ml) uptake using IncuCyte® live-cell analysis over 48 h. For each experiment cell death after 48 h of n=3 is shown (*p ≤ 0.05; **p ≤ 0.01; ***p ≤ 0.001; n.s. = not significant).

**Table S1. Related to Figure 4. Summary statistics of the sequencing reads and downstream analyses: trimming, mapping, and feature assignments.**

reads_total: number of raw single-end reads produced by the NextSeq500 machine

reads_retained: number of single-end reads after trimming of the raw reads (i.e trimmed reads)

avg_input_read_length: average length of trimmed single-end reads

uniquely_mapped: number of trimmed reads mapped uniquely by the STAR mapper

uniquely_mapped_percent: % of reads mapped uniquely by the STAR mapper from trimmed reads

assigned: number of uniquely mapped reads assign to a gene feature by featureCounts (RSubread)

assigned_percent: % of mapped reads assign to a gene feature from uniquely mapped reads

| Sample | condition | reads_  total | reads_  retained | avg_  input_  read_  length | uniquely_  mapped | uniquely_mapped_  percent | assigned | assigned  percent |
| --- | --- | --- | --- | --- | --- | --- | --- | --- |
| 1-1-1 | pA375 | 27046806 | 27045413 | 74 | 24420267 | 90,29 | 21904928 | 89,69 |
| 1-2-1 | pA375 | 27966065 | 27964180 | 74 | 24947278 | 89,21 | 22696004 | 90,97 |
| 1-3-1 | pA375 | 28681643 | 28679774 | 74 | 26237125 | 91,48 | 23894611 | 91,07 |
| 10-1-1 | pWM1346-IZI50 | 30174822 | 30171944 | 74 | 27297150 | 90,47 | 24975060 | 91,49 |
| 10-2-1 | pWM1346-IZI50 | 28145379 | 28143755 | 74 | 25289560 | 89,86 | 23050076 | 91,14 |
| 10-3-1 | pWM1346-IZI50 | 32069066 | 32067828 | 74 | 28675839 | 89,42 | 26391206 | 92,03 |
| 11-1-1 | cWM1346 | 27503858 | 27503310 | 74 | 24651103 | 89,63 | 22527944 | 91,38 |
| 11-2-1 | cWM1346 | 26780202 | 26778866 | 74 | 24055064 | 89,83 | 21786484 | 90,56 |
| 11-3-1 | cWM1346 | 35978165 | 35977080 | 74 | 32485310 | 90,29 | 29744521 | 91,56 |
| 12-1-1 | cWM1346-IZI50 | 26472392 | 26471426 | 74 | 23841064 | 90,06 | 21578580 | 90,51 |
| 12-2-1 | cWM1346-IZI50 | 32549788 | 32547156 | 74 | 29181361 | 89,66 | 26348538 | 90,29 |
| 12-3-1 | cWM1346-IZI50 | 29938961 | 29938178 | 74 | 26941183 | 89,99 | 24658153 | 91,52 |
| 2-1-1 | pA375-IZI50 | 27239624 | 27238855 | 74 | 24425440 | 89,67 | 21985451 | 90,01 |
| 2-2-1 | pA375-IZI50 | 22885504 | 22883731 | 74 | 20066006 | 87,69 | 18091472 | 90,15 |
| 2-3-1 | pA375-IZI50 | 28367545 | 28365500 | 74 | 25890666 | 91,28 | 23647092 | 91,33 |
| 3-1-1 | cA375 | 27712827 | 27712044 | 74 | 24924625 | 89,94 | 22299232 | 89,46 |
| 3-2-1 | cA375 | 30112564 | 30111907 | 74 | 26453438 | 87,85 | 23840146 | 90,12 |
| 3-3-1 | cA375 | 28612859 | 28610105 | 74 | 26196202 | 91,56 | 23813136 | 90,9 |
| 4-1-1 | cA375-IZI50 | 25724295 | 25722932 | 74 | 22217440 | 86,37 | 19897053 | 89,55 |
| 4-2-1 | cA375-IZI50 | 29075685 | 29067933 | 74 | 25365426 | 87,26 | 23057537 | 90,9 |
| 4-3-1 | cA375-IZI50 | 26223030 | 26218130 | 74 | 23332132 | 88,99 | 21276902 | 91,19 |
| 9-1-1 | pWM1346 | 24960502 | 24959365 | 74 | 22538772 | 90,3 | 20322364 | 90,16 |
| 9-2-1 | pWM1346 | 30458398 | 30457617 | 74 | 27548717 | 90,45 | 25052380 | 90,93 |
| 9-3-1 | pWM1346 | 32547495 | 32546209 | 74 | 29393654 | 90,31 | 26689498 | 90,8 |

All samples were sequenced with sufficient reads. More than 20 million of 74 bp in average were uniquely mapped on the human genome (minimum 20,066,006). Moreover, ~ 90% of these uniquely mapped reads were successfully assigned to gene features (Ensembl GRCh38 v100), leading to at least 18 million reads for counted features before differential gene expression analysis.

**Table S2. Related to Figure 4. Predicted common top regulators based on GeneWalk gene ontology analysis with at least 20 direct interactors within the underlying regulatory network.** Regulators are listed according to their function in “cell death/survival”, “migration/invasion”, “tumorigenesis”, and “other function”. Genes that showed a coherent regulation pattern in both A375 and WM1346 cells are shown on a white background, those that presented with opposing regulation were shaded in gray.

| **gene name** | **protein name – function in cell death/survival** | **PMID** |
| --- | --- | --- |
| MET | Mesenchymal-epithelial transition factor – RTK and proto-oncogene, mediating survival, migration and invasion through RAS-ERK and PI3K-AKT activation | [33066121](https://pubmed.ncbi.nlm.nih.gov/33066121/) |
| PML | PML Nuclear Body Scaffold - TRIM-family: transcription factor and tumor suppressor; regulates the p53 response to oncogenic signals | [33396222](https://pubmed.ncbi.nlm.nih.gov/33396222/) |
| PRKDC | DNA-dependent protein kinase (DNA-PK) catalytic subunit - DNA double strand break repair and recombination | [31380275](https://pubmed.ncbi.nlm.nih.gov/31380275/) |
| RARA | Retinoic acid receptor alpha - regulates cell growth arrest, differentiation, and apoptosis | [33069074](https://pubmed.ncbi.nlm.nih.gov/33069074/) |
| TRAF6 | tumor necrosis factor-associated factor 6 – survival via JNK/p38 and NFκB signaling | [33294443](https://pubmed.ncbi.nlm.nih.gov/33294443/) |
| **gene name** | **protein name – function in migration/invasion** | **PMID** |
| FAK(PTK2) | Focal adhesion kinase (protein tyrosine kinase 2) - cell migration, adhesion, spreading, reorganization of the actin cytoskeleton, formation and disassembly of focal adhesions and cell protrusions, cell cycle progression, cell proliferation and apoptosis | [27262114](https://pubmed.ncbi.nlm.nih.gov/27262114/) |
| IQGAP1 | Ras GTPase-activating-like protein – regulates cell adhesion, actin cytoskeleton, and cell cycle | [33434854](https://pubmed.ncbi.nlm.nih.gov/33434854/) |
| ITGAV | integrin alpha V subunit - regulates angiogenesis and cancer progression | [33605520](https://pubmed.ncbi.nlm.nih.gov/33605520/) |
| FN1 | Fibronectin 1 – ECM reprogramming in cancer, facilitating migration and invasion, promoting angiogenesis | [32426283](https://pubmed.ncbi.nlm.nih.gov/32426283/) |
| **gene name** | **protein name – function in tumorigenesis** | **PMID** |
| RB1 | RB Transcriptional Corepressor 1 - tumor suppressor regulation of entry into cell division by binding to E2F1 leading to cell cycle arrest. | [20637913](https://pubmed.ncbi.nlm.nih.gov/20637913/) |
| **gene name** | **protein name – other functions** | **PMID** |
| APP | Amyloid-Precursor-Protein - Alzheimer's disease | [33642366](https://pubmed.ncbi.nlm.nih.gov/33642366/) |
| EP300 | Histone acetyltransferase 300 – chromatin remodeling | [31965993](https://pubmed.ncbi.nlm.nih.gov/31965993/) |
| SCL11A2 | Solute carrier family 11 member 2 – transport of divalent metals and iron absorption | [16160008](https://pubmed.ncbi.nlm.nih.gov/16160008/) |
| CLTC | Clathrin Heavy Chain - major protein component of coated vesicles/pits | [30904808](https://pubmed.ncbi.nlm.nih.gov/30904808/) |
| HDAC4 | Histone deacetylase 4 - gene silencing | [33144118](https://pubmed.ncbi.nlm.nih.gov/33144118/) |
| SIRT1 | Sirtuin 1 - regulates epigenetic gene silencing | [33537003](https://pubmed.ncbi.nlm.nih.gov/33537003/) |
| SIRT7 | Sirtuin 7 – regulates epigenetic gene silencing | [33537003](https://pubmed.ncbi.nlm.nih.gov/33537003/) |
| SHTM2 | Serine Hydroxymethyltransferase 2 - mitochondrial: glycine synthesis | [33664451](https://pubmed.ncbi.nlm.nih.gov/33664451/) |

**Table S3. Related to Fig. 4. Log2FoldChanges and adjusted pvalues of FAK(PTK2) expression of untreated and 50 mg/ml IZI-treated (IZI) parental (p) and conditioned (c) A375 and WM1346 melanoma cells.**

|  | FAK(PTK2) | | | |
| --- | --- | --- | --- | --- |
|  | A375 | | WM1346 | |
| stimulation | log2FoldChange | pvalue | log2FoldChange | pvalue |
| c_vs_p | 0,398 | 0,0001 | 0,561 | 7,27E-08 |
| pIZI_vs_p | -0,557 | 7,05E-08 | -0,251 | 0,021 |
| cIZI_vs_c | -0,047 | 0,677 | -0,168 | 0,143 |
| cIZI_vs_pIZI | 0,894 | 9,85E-20 | 0,635 | 5,80E-10 |

**Table S4. Related to Figure 5. Direct interaction partners of FAK(PTK2) within the GeneWalk ontology-based regulatory network.** 64 direct interaction partners of FAK(PTK2) are listed according to their function in “cell death/survival”, “migration/invasion”, “tumorigenesis”, and “other function”. Genes that showed a common regulation pattern in both A375 and WM1364 cells are shown on a white background, those that presented with opposing regulation were shaded in gray.

| **gene name** | **protein name – function in cell death/survival** | **PMID** |
| --- | --- | --- |
| AKT3 | RAC-gamma serine/threonine-protein kinase 3 – proliferation, cell survival, growth, angiogenesis | [32120136](https://pubmed.ncbi.nlm.nih.gov/32120136/) |
| CDC14B | Cell Division Cycle 14B - Dual specificity phosphatase regulating G2 DNA checkpoint and p53 function | [20720150](https://pubmed.ncbi.nlm.nih.gov/20720150/) |
| DUSP6 | Dual-specificity phosphatase 6 – inactivates ERK | [31151270](https://pubmed.ncbi.nlm.nih.gov/31151270) |
| EYA4 | Transcriptional coactivator and phosphatase – DNA repair | [32727223](https://pubmed.ncbi.nlm.nih.gov/32727223) |
| IRF1 | Interferon regulatory factor 1 – TF, regulates DNA damage, apoptosis and tumor suppression | [28283576](https://pubmed.ncbi.nlm.nih.gov/28283576) |
| MET | Mesenchymal-epithelial transition factor – RTK and proto-oncogene, mediating survival, migration and invasion through RAS-ERK and PI3K-AKT activation | [33066121](https://pubmed.ncbi.nlm.nih.gov/33066121/) |
| PRKDC | DNA-dependent protein kinase (DNA-PK) catalytic subunit - DNA double strand break repair and recombination | [31380275](https://pubmed.ncbi.nlm.nih.gov/31380275/) |
| PTPN4 | Protein Tyrosine Phosphatase Non-Receptor Type 4 - cell-cell adhesion and growth control - prevents cell death induction in neuroblastoma and glioblastoma cell lines | [27246854](https://pubmed.ncbi.nlm.nih.gov/27246854) |
| PTRH2 | Peptidyl-TRNA hydrolase 2 - promotes survival as part of an integrin-signaling pathway in cells attached to the ECM, but promotes apoptosis in cells that have lost their ECM attachment | [33298880](https://pubmed.ncbi.nlm.nih.gov/33298880) |
| ATG7 | Autophagy related 7 – cytoplasmic to vacuole transport | [31211229](https://pubmed.ncbi.nlm.nih.gov/31211229) |
| CASP6 | Caspase-6 – apoptosis execution | [31675069](https://pubmed.ncbi.nlm.nih.gov/31675069) |
| DUSP10 | Dual-specificity phosphatase 10 – inactivates p38 and JNK | [31151270](https://pubmed.ncbi.nlm.nih.gov/31151270) |
| MAP3K5 | Apoptosis signal-regulating kinase 1 (ASK1) - activates MAPKs, JNKs, and p38 MAPKs, causing cell death and differentiation | [32362327](https://pubmed.ncbi.nlm.nih.gov/32362327) |
| RARA | Retinoic acid receptor alpha - regulates cell growth arrest, differentiation, and apoptosis | [33069074](https://pubmed.ncbi.nlm.nih.gov/33069074) |
| RNF34 | Ring Finger Protein 34 - anti-apoptotic E3 ubiquitin-protein ligase - ubiquitinates the caspases-8 and -10, RIPK1, and p53 - caspase-3 | [15069192](https://pubmed.ncbi.nlm.nih.gov/15069192) |
| **gene name** | **protein name – function in migration/invasion** | **PMID** |
| ABLIM1 | Actin binding LIM protein 1 – cytoskeletal organization | [23974990](https://pubmed.ncbi.nlm.nih.gov/23974990) |
| ARHGAP26 | Rho GTPase activating protein 26 – integrin-mediated organization of the cytoskeleton via FAK | [31332482](https://pubmed.ncbi.nlm.nih.gov/31332482) |
| ASAP1 | Arf-GAP with SH3 domain, ANK repeat and PH domain-containing protein 1 – membrane trafficking and cytoskeleton remodeling | [30669557](https://pubmed.ncbi.nlm.nih.gov/30669557) |
| COL13A1 | Collagen alpha-1(XIII) chain – cell-cell and cell-matrix interaction | [29874875](https://pubmed.ncbi.nlm.nih.gov/29874875) |
| GIT1 | GTPase-activating protein – present in integrin adhesion complexes, regulates survival, proliferation, migration | [28362242](https://pubmed.ncbi.nlm.nih.gov/28362242) |
| ICAM1 | Intercellular adhesion molecule 1 - stabilizing cell-cell interactions | [33553361](https://pubmed.ncbi.nlm.nih.gov/33553361) |
| IQGAP1 | Ras GTPase-activating-like protein – regulates cell adhesion, actin cytoskeleton, and cell cycle | [33434854](https://pubmed.ncbi.nlm.nih.gov/33434854) |
| ITGA3 | intergrin alpha 3 subunit - may be correlated with breast cancer metastasis. | [33605520](https://pubmed.ncbi.nlm.nih.gov/33605520) |
| ITGA4 | integrin alpha 4 subunit - cell motility and migration | [33605520](https://pubmed.ncbi.nlm.nih.gov/33605520) |
| ITGA6 | integrin alpha 6 subunit - promotes tumorigenesis | [33605520](https://pubmed.ncbi.nlm.nih.gov/33605520) |
| ITGAV | integrin alpha V subunit - regulates angiogenesis and cancer progression | [33605520](https://pubmed.ncbi.nlm.nih.gov/33605520) |
| LAMA4 | Laminin subunit alpha4 - cell adhesion, differentiation, migration, signaling, and metastasis | [33605520](https://pubmed.ncbi.nlm.nih.gov/33605520) |
| LAMB1 | Laminin subunit beta 1 - cell adhesion, differentiation, migration, signaling, and metastasis | [33605520](https://pubmed.ncbi.nlm.nih.gov/33605520) |
| LAMC1 | Laminin subunit gamma 1 - cell adhesion, differentiation, migration, signaling, and metastasis | [33605520](https://pubmed.ncbi.nlm.nih.gov/33605520) |
| LIMS1 | LIM Zinc Finger Domain Containing 1 - adaptor protein involved in integrin-mediated cell adhesion or spreading | [17084981](https://pubmed.ncbi.nlm.nih.gov/17084981) |
| COL6A1 | Collagen alpha-1(VI) chain – tissue integrity | [29277723](https://pubmed.ncbi.nlm.nih.gov/29277723) |
| FN1 | Fibronectin 1 – ECM reprogramming in cancer, facilitating migration and invasion, promoting angiogenesis | [32426283](https://pubmed.ncbi.nlm.nih.gov/32426283) |
| NEO1 | Neogenin 1 - cell growth, differentiation and cell-cell adhesion | [32117748](https://pubmed.ncbi.nlm.nih.gov/32117748) |
| **gene name** | **protein name – function in tumorigenesis** | **PMID** |
| ABCC4 | ATP-binding cassette sub-family C member 4 – multi drug resistance | [30123371](https://pubmed.ncbi.nlm.nih.gov/30123371) |
| RB1 | RB Transcriptional Corepressor 1 - tumor suppressor regulation of entry into cell division by binding to E2F1 leading to cell cycle arrest. | [20637913](https://pubmed.ncbi.nlm.nih.gov/20637913) |
| CRK | Proto-oncogene c-Crk – SH2/SH3 domain-containing adapter to modulate RTK signaling | [21917713](https://pubmed.ncbi.nlm.nih.gov/21917713) |
| CRKL | Crk-like protein - SH2/SH3 domain-containing adapter to modulate RTK signaling | [26554907](https://pubmed.ncbi.nlm.nih.gov/26554907) |
| HBEGF | Heparin-binding EGF-like growth factor – cell cycle progression, cell survival, adhesion, and cell migration | [27635238](https://pubmed.ncbi.nlm.nih.gov/27635238) |
| NF1 | Neurofibromin 1 - negative regulator of Ras signal transduction | [31683701](https://pubmed.ncbi.nlm.nih.gov/31683701) |
| PTPRM | Protein tyrosine phosphatase receptor type M - cell growth, differentiation, mitotic cycle, and oncogenic transformation | [25234613](https://pubmed.ncbi.nlm.nih.gov/25234613) |
| RASA1 | RAS P21 Protein Activator 1 - stimulates GTPase activity of normal but not oncogenic RAS p21; controls proliferation and differentiation | [32717309](https://pubmed.ncbi.nlm.nih.gov/32717309) |
| **gene name** | **protein name – other functions** | **PMID** |
| APP | Amyloid-Precursor-Protein - Alzheimer's disease | [33642366](https://pubmed.ncbi.nlm.nih.gov/33642366) |
| CPD | Carboxypeptidase D - biosynthesis of neuropeptides and peptide hormones | [26523264](https://pubmed.ncbi.nlm.nih.gov/26523264) |
| EP300 | Histone acetyltransferase 300 – chromatin remodeling | [31965993](https://pubmed.ncbi.nlm.nih.gov/31965993) |
| HSD17B4 | Hydroxysteroid 17-Beta Dehydrogenase 4 – fatty acids elongation | [30508570](https://pubmed.ncbi.nlm.nih.gov/30508570) |
| MT2A | Metallothionein 2A – modulates concentration of heavy metals | [27608012](https://pubmed.ncbi.nlm.nih.gov/27608012) |
| PPCDC | Phosphopantothenoylcysteine Decarboxylase - Biosynthesis of coenzyme A (CoA) from pantothenic acid (vitamin B5) | [15450493](https://pubmed.ncbi.nlm.nih.gov/15450493) |
| SCL11A2 | Solute carrier family 11 member 2 – transport of divalent metals and iron absorption | [16160008](https://pubmed.ncbi.nlm.nih.gov/16160008) |
| SLC23A2 | Solute carrier family 23 member 2 - tissue-specific vitamin C uptake | [32751086](https://pubmed.ncbi.nlm.nih.gov/32751086) |
| ATP7A | Menkes' protein (MNK) – copper transport across membranes | [33482423](https://pubmed.ncbi.nlm.nih.gov/33482423) |
| CAT | Catalase – defense against oxidative stress | [33621439](https://pubmed.ncbi.nlm.nih.gov/33621439) |
| CPS1 | Carbamoyl phosphatase synthetase 1 – urea cycle | [33317798](https://pubmed.ncbi.nlm.nih.gov/33317798) |
| DNAJC6 | DNAJ/HSP40 family of proteins - Parkinson's Disease | [31120186](https://pubmed.ncbi.nlm.nih.gov/31120186) |
| ESRRA | Estrogen-related receptor alpha – TF: cellular energy production | [30719023](https://pubmed.ncbi.nlm.nih.gov/30719023) |
| FADS1 | Fatty acid desaturase 1 - unsaturation of fatty acids | [31433740](https://pubmed.ncbi.nlm.nih.gov/31433740) |
| GNAS | G-protein subunit alpha – signal transduction | [33574795](https://pubmed.ncbi.nlm.nih.gov/33574795) |
| GPX1 | Glutathione peroxidase 1 - detoxification of hydrogen peroxide | [31300958](https://pubmed.ncbi.nlm.nih.gov/31300958) |
| HSD17B12 | Hydroxysteroid 17-Beta Dehydrogenase 12 – fatty acids elongation | [30508570](https://pubmed.ncbi.nlm.nih.gov/30508570) |
| PDE4B | cAMP-specific 3',5'-cyclic phosphodiesterase 4B - break down cyclic nucleotides (cAMP, cGMP) mediated signal transduction | [32203163](https://pubmed.ncbi.nlm.nih.gov/32203163) |
| PPP1CB | Protein Phosphatase 1 (PP1) Catalytic Subunit Beta - regulation of several cellular functions, including cell division | [25620225](https://pubmed.ncbi.nlm.nih.gov/25620225) |
| REPIN1 | Replication Initiator 1 - initiation of chromosomal DNA replication | [23374714](https://pubmed.ncbi.nlm.nih.gov/23374714) |
| SIRT1 | Sirtuin 1 - regulates epigenetic gene silencing | [33537003](https://pubmed.ncbi.nlm.nih.gov/33537003) |
| SMOX | Spermine Oxidase - alters polyamine homeostasis | [31207342](https://pubmed.ncbi.nlm.nih.gov/31207342) |

**Table S5. Related to Fig. 5. Log2FoldChanges and adjusted p-values of IQGAP1, ITGAV, PRKDC, and MET expression of untreated and 50 mg/ml IZI-treated (IZI) parental (p) and conditioned (c) A375 and WM1346 melanoma cells.**

|  | IQGAP1 | | | |
| --- | --- | --- | --- | --- |
|  | A375 | | WM1346 | |
| stimulation | log2FoldChange | pvalue | log2FoldChange | pvalue |
| c_vs_p | -0,466 | 4,32E-06 | 0,106 | 0,592 |
| pIZI_vs_p | -0,772 | 1,08E-14 | -0,575 | 2,43E-06 |
| cIZI_vs_c | 0,059 | 0,615 | -0,226 | 0,0085 |
| cIZI_vs_pIZI | 0,385 | 1,60E-04 | 0,448 | 4,27E-04 |

|  | ITGAV | | | |
| --- | --- | --- | --- | --- |
|  | A375 | | WM1346 | |
| stimulation | log2FoldChange | pvalue | log2FoldChange | pvalue |
| c_vs_p | 0,294 | 0,172 | -3016E-04 | 0,998 |
| pIZI_vs_p | -0,384 | 0,034 | -0,588 | 6,00E-04 |
| cIZI_vs_c | 0,040 | 0,745 | 0,115 | 0,468 |
| cIZI_vs_pIZI | 0,832 | 2,48E-06 | 0,766 | 8,27E-06 |

|  | PRKDC | | | |
| --- | --- | --- | --- | --- |
|  | A375 | | WM1346 | |
| stimulation | log2FoldChange | pvalue | log2FoldChange | pvalue |
| c_vs_p | 0,198 | 0,349 | 0,075 | 0,770 |
| pIZI_vs_p | -0,983 | 7,74E-10 | -0,828 | 1,72E-12 |
| cIZI_vs_c | 0,021 | 0,878 | -0,011 | 0,938 |
| cIZI_vs_pIZI | 1,258 | 3,92E-16 | 0,907 | 1,16E-14 |

|  | MET | | | |
| --- | --- | --- | --- | --- |
|  | A375 | | WM1346 | |
| stimulation | log2FoldChange | pvalue | log2FoldChange | pvalue |
| c_vs_p | 0,284 | 0,036 | 0,303 | 0,020 |
| pIZI_vs_p | -0,608 | 5,53E-07 | -0,443 | 1,42E-04 |
| cIZI_vs_c | 0,131 | 0,332 | -0,108 | 0,396 |
| cIZI_vs_pIZI | 1,111 | 4,99E-22 | 0,644 | 1,96E-08 |

**Table S6. Related to Fig. 6. Log2FoldChanges and adjusted p-values of BAK, BAX, and PUMA expression of untreated and 50 mg/ml IZI-treated (IZI) parental (p) and conditioned (c) A375 and WM1346 melanoma cells.**

|  | BAK | | | |
| --- | --- | --- | --- | --- |
|  | A375 | | WM1346 | |
| stimulation | log2FoldChange | pvalue | log2FoldChange | pvalue |
| c_vs_p | 0,961 | 5,89E-07 | -0,133 | 0,587 |
| pIZI_vs_p | 1,135 | 4,146E-09 | 1,005 | 4,19E-10 |
| cIZI_vs_c | 0,123 | 0,375 | 0,700 | 1,08E-04 |
| cIZI_vs_pIZI | 0,102 | 0,681 | -0,434 | 0,009 |

|  | BAX | | | |
| --- | --- | --- | --- | --- |
|  | A375 | | WM1346 | |
| stimulation | log2FoldChange | pvalue | log2FoldChange | pvalue |
| c_vs_p | 0,331 | 0,160 | -0,124 | 0,532 |
| pIZI_vs_p | 0,190 | 0,306 | 0,429 | 5,46E-04 |
| cIZI_vs_c | 0,122 | 0,376 | 0,482 | 4,09E-04 |
| cIZI_vs_pIZI | 0,403 | 0,056 | -0,074 | 0,661 |
|  | PUMA(BBC3) | | | |
|  | A375 | | WM1346 | |
| stimulation | log2FoldChange | pvalue | log2FoldChange | pvalue |
| c_vs_p | 0,332 | 0,404 | 0,029 | 0,892 |
| pIZI_vs_p | 0,036 | 0,908 | 0,300 | 0,328 |
| cIZI_vs_c | 0,015 | 0,882 | -0,074 | 0,309 |
| cIZI_vs_pIZI | 0,384 | 0,275 | -0,783 | 0,066 |
